# An integrated Msr-antioxidase-host gene circuit maintains redox homeostasis in legume-rhizobium symbiosis

**DOI:** 10.3389/fpls.2026.1811549

**Published:** 2026-06-03

**Authors:** Zaiyong Si, Yuxin Wang, Meng Shen, Yanxia Yu, Feng Wei, Yuxun Lu, Xiufeng Long, Yi Yi, Hui Lin, Youguo Li

**Affiliations:** 1 Guangxi Key Laboratory for Green Processing of Sugar Resources, College of Biological and Chemical Engineering, Guangxi University of Science and Technology, Liuzhou, China; 2 National Key Laboratory of Agricultural Microbiology, College of Life Science and Technology, Huazhong Agricultural University, Wuhan, China; 3 Oil Crops Research Institute, Chinese Academy of Agricultural Sciences, Wuhan, China

**Keywords:** host-microbe interaction, methionine sulfoxide reductase (Msr), oxidative stress, redox homeostasis, symbiotic nitrogen fixation

## Abstract

Methionine sulfoxide reductases (Msrs) play a critical role in oxidative stress resistance; however, their functions in rhizobium-legume symbiotic nitrogen fixation (SNF) are not well understood. In this study, we systematically characterized four Msrs (MsrA1, MsrB1, MsrA2, MsrB2) from *Mesorhizobium huakuii* 7653R, a symbiotic partner of *Astragalus sinicus*. Sequence and phylogenetic analyses confirmed the presence of conserved catalytic domains and revealed genus-specific clustering of these Msrs. Expression profiling demonstrated distinct patterns: *msrA1* and *msrB1* were transiently induced during early symbiotic infection, whereas *msrA2* and *msrB2* exhibited biphasic upregulation at both early infection and nodule maturation stages. Notably, *msrA1* responded specifically to H_2_O_2_, and all *msr* genes were induced by sodium hypochlorite in a concentration-dependent manner. Phenotypic analyses of overexpression (OE) and deletion (Δ) strains indicated that Msrs modulate key bacterial physiological traits. Deletion mutants showed impaired motility, reduced biofilm formation, and decreased activities of antioxidant enzymes (catalase, glutathione peroxidase, superoxide dismutase), accompanied by elevated intracellular superoxide anion and H_2_O_2_ content. In contrast, *msrs* overexpression enhanced oxidative stress resistance but suppressed bacterial growth. In symbiotic assays, overexpression of *msrA1*, *msrA2*, or *msrB2* resulted in leaf chlorosis, reduced nodule number, and impaired nitrogen fixation efficiency, while *msrA2Δ* and *msrB2Δ* mutants affected nodulation without compromising plant vigor. Further investigation revealed that Msrs regulate host root antioxidant responses and the transcription of symbiotic-related genes (*AsNIN*, *AsNPL2*) and defense-related genes (*AsFLS2*, *AsPR10*). Bacterial two-hybrid assays identified physical interactions between Msrs and chaperone proteins (GroEL1/2/3), antioxidant enzymes (SodA/B, KatE/G), and the LysR-type transcriptional regulator LsrB, suggesting the formation of an integrated redox regulatory network. Collectively, our findings demonstrate functional specialization of Msrs in *M. huakuii* 7653R, mediating oxidative stress resistance, bacterial physiology, and host–symbiont crosstalk. We propose a “Msr – antioxidant enzyme – host gene” regulatory model that maintains redox homeostasis during SNF. This study provides novel insights into the roles of rhizobial Msrs and offers potential targets for engineering high-efficiency nitrogen-fixing strains.

## 1 Introduction

Reactive oxygen species (ROS), including hydrogen peroxide (H_2_O_2_), superoxide anion (O^2-^) and hydroxyl radical (HO^•^), are generated during the establishment of rhizobium-legume symbiosis (Peleg-Grossman et al., 2012). The oxidation of biological macromolecules such as nucleic acid, lipid and protein by ROS will disrupt their structure or function (Beckman and Ames, 1997). To protect against ROS, a series of anti-oxidant devices have been selected throughout evolution. Aerobic organisms have evolved both enzymatic and nonenzymatic scavenging systems. The enzymatic systems include superoxide dismutases, catalases, peroxidases and methionine sulfoxide reductase (Msr). In protein, the sulfur-containing amino acids methionine is readily oxidized by ROS to form two stereoisomeric forms of MetO (*R*-MetO and *S*-MetO), which are restored to methionine by methionine sulfoxide reductase A (MsrA) and methionine sulfoxide reductase B (MsrB), respectively (Gao et al., 1998; Vincent and Ezraty, 2022). The oxidation and reduction of methionine is a common phenomenon in biological systems under both physiological and oxidative stress conditions, and methionine sulfoxide is involved in multiple signal transduction pathways that are crucial for cellular function (Moskovitz and Smith, 2021). Although MsrA and MsrB are functionally similar, their sequences are quite different, the amino acid sequences of MsrA contained a conserved sequence (GCFWG) located at the N-terminal portion of the protein and the cysteine in this sequence has been shown to be essential for enzymatic activity (Moskovitz et al., 2000; Fukushima et al., 2007). The amino acid sequences of MsrB are classified into two groups by the presence (form I) or absence (form II) of two CxxC motifs that have been proved to participate in binding to a divalent zinc cation (Ding et al., 2007).

Msrs are widely present in bacteria, yeast, mammals and plants, and play a significant role in the interaction between pathogens and the host (Josenhans and Suerbaum, 2002; Romsang et al., 2013; Li et al., 2017; Sarkhel et al., 2017; Vanhove et al., 2017; Singh et al., 2018; Maniraj et al., 2025; Su-Ming et al., 2025). In pathogenic bacteria, Msrs affect the infection ability, survival ability and virulence by altering their adhesion, motility, oxidation resistance and biofilm formation (Santos et al., 2001; Beloin et al., 2004; Kuboniwa et al., 2006). For example, *msrA1*-deficient *Staphylococcus aureus* exhibited a significant decrease in adhesion ability to human lung epithelial cells (Singh et al., 2015). Motility is an important determinant of bacterial virulence, which can help the evasion of bacteria to the host (Ottemann and Miller, 1997; Josenhans and Suerbaum, 2002). For instance, *msrA*-deletion *Erwinia chrysanthemi* showed a weakened motility on the plate, resulting in poorer ability of infection plants (Hassouni et al., 1999). In addition, *msrA*-deletion *Mycoplasma genitalium* could not colonize in hamster lungs (Dhandayuthapani et al., 2001). The deletion of *msrA* or *msrB* in *Helicobacter pylori* markedly decreased its colonization number in the large intestine of mice (Alamuri and Maier, 2004). *Pseudomonas aeruginosa* with the deletion of *msrA* and/or *msrB* became more sensitive to sodium hypochlorite and hydrogen peroxide with an obvious decrease in virulence (Romsang et al., 2013). The above findings indicate that *msrs* play a positive role in pathogens for invasion of their host.

Identification of the substrate proteins for Msrs is conducive to clarify their functional mechanism. In *H. pylori*, three interacting substrates of Msrs, molecular partner GroEL, peroxidase KatA and thioredoxin Trx1, were screened by affinity chromatography and they participated in antioxidant stress (Alamuri and Maier, 2006). GroEL is inactivated once oxidized by hypochlorite, but its activity can be recovered after incubation with MsrA/B (Khor et al., 2004). MsrA can repair the activity of malate synthetase oxidized by hydrogen peroxide in *Salmonella typhimurium* (Sarkhel et al., 2017). Recently, it was reported that MsrA could recover the activity of oxidized recombinant enzyme RecA in *E. coli* (Speck et al., 2019). Mxr2 interacts with the two core proteins of the cytoplasm to vacuole targeting (Cvt) autophagy pathway *in vivo*, Atg19 and Ape1, to resist oxidative stress (Chatterjee and Sepuri, 2024). These studies indicate that Msrs are involved in many life activities by interacting with substrate proteins.

Many genes participate in regulating the balance of redox state during the symbiotic nitrogen fixation process (Ribeiro et al., 2015). Two superoxide dismutases (SodA and SodB) (Santos et al., 2000) and three peroxidases (KatA, KatB and KatC) (Ardissone et al., 2004) are present in *S. meliloti*. The expression of 2-cysteine peroxidase (Prxs) is induced at the early stage of symbiotic nitrogen fixation (Baron and Z., 1995). Furthermore, chloride peroxidase is secreted by rhizobia when exposed to hydrogen peroxide and organic hydroperoxides (Baron and Z., 1995). The transcription factor lsrB, which belongs to the LysR family, is necessary for regulating the expression of antioxidant gene in *S. meliloti* 1021 (Tang et al., 2017). The activity of HypT could be activated by sodium hypochlorite oxidation, leading to the expression of the downstream antioxidant gene in *E. coli*, but would be decreased when its methionine was restored by MsrA and MsrB (Drazic et al., 2013). The results of homologous sequence alignment show that LsrB in *Mesorhizobium huakuii* 7653R is the orthologous protein of HypT in *E. coli*.

The functional mechanism of Msrs is increasingly clarified in the interaction between pathogens and the host (Romsang et al., 2013; Li et al., 2017; Sarkhel et al., 2017; Vanhove et al., 2017; Singh et al., 2018; Zhou et al., 2024). However, the functional mechanism of Msrs in rhizobia-legume symbiosis remains unclear. *Astragalus sinicus* (Chinese milk vetch) is a winter-growing green manure legume and can form indeterminate-type nitrogen-fixing nodules with *M. huakuii* (Chen et al., 1991). A methionine sulfoxide reductase gene has been previously identified from *Astragalus sinicus* (*AsMsrB*), which was found to be necessary for the establishment of symbiotic nitrogen fixation process (Wang et al., 2019). We have identified four *msrs* (two *msrAs* and two *msrBs*) from *M. huakuii* 7653R.

Here, we first elucidate the functional roles of rhizobial Msrs in resisting oxidative stress during symbiosis. To explore their effects on bacterial physiological traits and symbiotic capacity, we constructed overexpression and deletion strains of four *msr* genes. Physiological assays demonstrated that Msrs regulate bacterial growth, oxidative stress tolerance, antioxidant enzyme activities, motility, biofilm formation, as well as the accumulation of intracellular superoxide anions and H_2_O_2_. Symbiotic assays with *Astragalus sinicus* further verified that Msrs modulate nodulation phenotypes, host root antioxidant status, and the expression of symbiosis- and defense-related genes. In addition, three types of antioxidant proteins were identified as interaction partners of Msrs. Collectively, our findings highlight that Msrs are essential for symbiosis establishment by fine-tuning the bacterial antioxidant system and precisely regulating host symbiotic and defense gene expression. This work offers novel mechanistic insights into oxidative stress adaptation in rhizobia during symbiotic nitrogen fixation.

## 2 Materials and methods

### 2.1 Characterization and phylogenetic analysis

All the MsrA and MsrB sequences were obtained from the NCBI database (https://www.ncbi.nlm.nih.gov/). Conserved domain analysis was performed on the Protein BLAST (https://blast.ncbi.nlm.nih.gov/Blast.cgi). All amino acid sequence alignments were conducted with the DNAMAN software (https://dnaman-32-en-001.software.informer.com/). The multi-sequence alignment was performed using Clustal W (https://www.ddbj.nig.ac.jp/services/clustalw-e.html) and the phylogenetic trees were constructed with the Neighbor-Joining method of MEGA 6.0 software (http://www.megasoftware.net/).

### 2.2 Plant materials and growth conditions

The *A. sinicus* seeds were collected from Xinyang, Henan province and treated according to the method previously reported (Cho et al., 1998). The seedlings were planted in sterilized sands, watered with Nitrogen-Free Solution (NFS) (F., 1957) and inoculated with *M. huakuii* 7653R, native promoter strains or deletion mutant strains upon the appearance of cotyledons. All plants were grown under controlled conditions (22 °C/25 °C and 16-h/8-h light/dark cycles). Symbiotic phenotype was examined 4–5 weeks after inoculation with rhizobia.

### 2.3 Construction of native promoter and deletion mutant strains

To construct *msr* native promoter strains, four fragments, *msrA1*, *msrB1*, *msrA2* and *msrB2*, containing their promoters and open reading frames (ORFs), were amplified, respectively. The PCR products were double digested with *Eco*R I and *Bam*H I, and cloned into pBBR1MCS-5 plasmid (Gene ID: U25061) and firstly transformed into *E. coli* S17-1. The empty plasmid pBBR1MCS-5 was used as the control. All the recombinant plasmids were transferred into *M. huakuii* 7653R by the combination of two parents. The native promoter strains were verified by PCR amplification with universal primer PBBR-F/R, which are the sequence matching on both sides of the pBBR1MCS-5 cloning site, and the PCR products were sequenced. All the primers are listed in **Supplementary Table 1**.

*Msrs* deletion mutants were constructed with the *Cre*-*lox* system (Marx and L., 2002). The specific steps were as follow*s* and the construction processes were listed in **Supplementary Figures 1A–E**). The flanks upstream and downstream fragments (about 600 bp) of *msrA1*, *msrB1*, *msrA2* and *msrB2* were amplified and sequenced, respectively. The fragments were double digested with corresponding restriction endonuclease. The flanks fragments of *msrA1*, *msrA2* and *msrB2* were cloned into the vector PCM351, and the flanks fragments of *msrB1* were cloned into the vector PCM184. All primers used are listed in **Supplementary Table 1**. The recombinant plasmids were firstly transformed into *E. coli* S17-1, and then transferred into *M. huakuii* 7653R by the combination of two parents. The transformation products of *msrA1*, *msrA2* and *msrB2* were diluted and coated on the acid minimal salts (AMS) medium with streptomycin and gentamycin, and the transformation products of *msrB1* were diluted and coated on the screening medium AMS medium with streptomycin and kanamycin. AMS medium derived from the literature, with some changes. The detailed recipe and ingredients for AMS medium are provided in the “Acid Minimal Salts (AMS) Medium” section of the **Supplementary Data**. All strains grown at 28 °C, and the exchange strains were obtained. The resistant gene on the plasmid replaces the target gene by homologous recombination. The plasmid PCM157 or PCM158 with the expression of the recombinant enzyme *Cre* was transformed into the exchange strains to delete the resistant gene. Finally, the deletion mutants were verified by PCR using corresponding map primers and those PCR products were sequenced. And different strains were cultured with ampicillin (Amp, 100 μg/mL), streptomycin (Str, 50 μg/mL), gentamycin (Gm, 25 μg/mL), kanamycin (Kan, 50 μg/mL), and tetracyclines (Tet, 12.5 μg/mL), respectively.

### 2.4 Bacterial strains and growth conditions

*M. huakuii* 7653R, native promoter strains and deletion mutant strains were grown at 28°C in the Tryptone-Yeast extract (TY) medium, and *E. coli* cells were grown at 37°C in Luria-Bertani broth. *E. coli* XL1-Blue MRF’, the host strain for Bacterio Match II two-hybrid analysis, and its derivatives were grown according to the manufacturer’s instructions (Stratagene). Different strains were cultured with ampicillin (100 μg/mL), streptomycin (50 μg/mL), gentamycin (25 μg/mL), kanamycin (50 μg/mL), and tetracyclines (12.5 μg/mL), respectively.

### 2.5 Determination of antioxidant enzyme activity

The activities of three antioxidant enzymes (catalase, glutathione peroxidase, and superoxide dismutase) were measured according to the instructions provided by the corresponding reagent kits (Beijing Boxbio Science & Technology Co., Ltd.). For bacterial samples, cultures were shaken until reaching an OD_600_ of 0.6. Subsequently, 10 mL of bacterial solution was collected and centrifuged at 5,000 rpm for 5 min at room temperature. The supernatant was discarded, and the cell pellet was resuspended in the protein extraction buffer included in the kit, followed by thorough disruption using a high-pressure homogenizer. The resulting lysate was centrifuged at 8,000 ×g for 10 min at 4 °C to obtain the crude enzyme extract. For root samples of *Astragalus sinicus*, fresh roots were collected at 3, 5 and 7 days post-inoculation. The tissues were ground to a fine powder in liquid nitrogen and centrifuged to obtain the enzyme extract, which was then assayed according to the kit protocol. In all assays, enzyme activities were determined following the manufacturer’s procedures. Each experiment was performed in three independent replicates.

### 2.6 Motility assay of rhizobium

Bacterial motility was evaluated using a modified soft-agar assay based on previously described protocols (Simon et al., 2015). Strains were first activated on TY agar plates at 28 °C for 48 h. A single colony was then inoculated into 5 mL of TY liquid medium containing the appropriate antibiotics and grown at 28 °C with shaking (200 rpm) for 48 h. The optical density at 600 nm (OD_600_) of each culture was measured and adjusted to approximately 0.5 with sterile TY medium. A 2 μL aliquot of the adjusted culture was spotted onto 20% TY semi-solid agar plates, with three biological replicates per strain. Plates were incubated at 28 °C for 72 h, after which images were captured, and the diameters of the motility zones were measured using GIMP software (version 2.10). The expansion area on soft agar was used as a quantitative indicator of bacterial motility.

### 2.7 Biofilm formation assay of rhizobium

Biofilm formation was assessed according to a previously reported method with slight modifications (M. et al., 2012). In brief, rhizobial strains were first revived on TY agar plates at 28 °C for 48 h. Single colonies were then inoculated into 5 mL of TY liquid medium containing appropriate antibiotics and cultured at 28 °C with shaking at 200 rpm for 48 h. Cells were harvested by centrifugation at 6000 rpm for 5 min and washed three times with 30% MM medium. The resulting cell suspension was adjusted to an OD_600_ of 0.2. A 100 µL aliquot of the adjusted suspension was transferred into each well of a sterile polystyrene 96 well plate, with three replicates per strain, and incubated statically at 28 °C for 72 h. After incubation, planktonic cells were removed and the wells were gently washed twice with deionized water. Attached biofilms were stained with 200 µL of 0.1% crystal violet for 30 min, followed by three washes with deionized water. The bound dye was dissolved in 100 µL of 80% ethanol–20% acetone mixture, and absorbance was measured at 570 nm using a microplate reader. Biofilm formation was quantified as the A_570_/OD_600_ ratio, with higher values indicating greater biofilm production.

### 2.8 Determination of the intracellular superoxide anion and hydrogen peroxide

In this study, determination of the intracellular superoxide anion content were measured according to the instructions provided by the corresponding reagent kits (Beijing Boxbio Science & Technology Co., Ltd.). The fluorescent sensor HYTZ-1 were employed to determinate hydrogen peroxide, HYTZ-1 can specifically recognize hydrogen peroxide and realize a fast and specific response toward H_2_O_2_ (Luary et al., 2014). For hydrogen peroxide detection in rhizobia, bacterial cultures were adjusted to an optical density (OD) of approximately 0.6 before adding the fluorescent probe HYTZ-1. For hydrogen peroxide measurement in root tissues, samples were collected at different time points after rhizobial inoculation, immediately ground in liquid nitrogen, and homogenized in extraction buffer. After centrifugation, the supernatant was incubated with the HYTZ-1 probe. In both cases, the reaction was allowed to proceed for 15 min, and fluorescence intensity was measured using a microplate reader. The relative fluorescence intensity was used to represent hydrogen peroxide content.

### 2.9 Determination of oxidative sensitivity by filter paper disc method

First, freshly cultured rhizobia were inoculated into liquid TY medium containing antibiotics and incubated at 28 °C with shaking at 220 rpm for 24–32 h. The bacterial suspension was then adjusted to an OD_600_ of 0.5. Next, double-layer agar plates were prepared: the bottom layer consisted of standard TY agar, and the top layer was composed of 0.8% agar TY medium mixed with the adjusted bacterial suspension, which was poured over the solidified bottom layer. Sterilized filter paper discs (6 mm in diameter) were immersed in oxidant solutions at various concentrations. After removal, excess liquid was gently absorbed, and the discs were placed onto the surface of the double-layer plates. Finally, the plates were inverted and incubated at 28 °C for 2–3 days. The inhibition zones were photographed, and their diameters were measured using the cross method (by measuring two perpendicular diameters).

### 2.10 RNA extraction and real-time PCR

Under the free-living and symbiotic conditions, all the strains (OD_600_ 0.6–0.8) were collected and the roots and nodules at different days after inoculation with *M. huakuii* 7653R were collected, respectively. To test the expression characteristics of *msrAs* and *msrBs* in response to oxidative stress. *M. huakuii* 7653R was transferred to 100 mL TY medium (V/V = 1%) at 28 °C under 220 rpm shaking for 2–3 days (OD_600_ 0.6–0.8), followed by the addition of hydrogen peroxide to reach the final concentration of 0, 1, 5 and 10 mM, or sodium hypochlorite to reach the final concentration of 0%, 0.005%, 0.01% and 0.05%, and treated for 30 min at 28 °C and 220 rpm. *M. huakuii* 7653R was collected by centrifugation at 12–000 rpm for 1 min.

Total RNA was extracted from each sample using the Trizol reagent (Aidlab) according to the manufactures’ instructions, we firstly removed genomic DNA and then performed reverse transcription. The cDNA was synthesized by RevertAid Reverse Transcription (Fermentas) using random primers. Real-time PCR was performed using the Fast Start Universal SYBR Green Master Kit (Roche), and the fluorescence signals were monitored and analyzed using the Applied Biosystems (ABI) StepOne. The transcript levels of *msrA1*, *msrB1*, *msrA2* and *msrB2* were examined, and RNase P RNA gene (*rnpB*) was used as the internal reference. All real-time PCR primers were listed in **Supplementary Table 1**. Relative gene expression was quantified using the 2^−ΔΔCt^ method. The transcript level of *msr* genes in free-living wild-type 7653R was set as the reference (value = 1.0), with *rnpB* used as the internal reference gene. All expression results are presented as the fold change of *msr* transcription in samples from different time points and nodule tissues relative to free-living wild-type 7653R. Each experiment was repeated three times.

### 2.11 Nitrogenase activity measurement and paraffin section observation

Determination of nitrogenase activity was performed with the acetylene reduction activity method (Hardy et al., 1973). Nine plants were collected from each experimental group. The underground part of three plants was put in a bottle with 2 mL acetylene, allowed to react at 28°C for 3 h, and then 100 μL of gas was collected and analyzed by GC 4000A gas chromatography (East & West analytical instrument).

Nodules were collected at 30 dpi and cut longitudinally, and then fixed in FAA buffer, washed for two or three times, dehydrated and then embedded in paraffin. Finally, the slides were stained with toluidine blue. The paraffin section was observed by the Olympus light microscope.

### 2.12 The detailed prediction method of candidate proteins

Candidate proteins of four Msrs were predicted at the protein interaction prediction website (https://string-db.org/). The thorough prediction steps were descripted in the **Supplementary Data** (**Supplementary Figures 2**-**S4**). Finally, interacting proteins were obtained and the results were listed in the Review-only **Supplementary Materials**.

### 2.13 Bacterial two-hybrid assays

Analysis of protein-protein interactions between MsrA/B and candidate proteins was conducted with the BacterioMatchII Two-Hybrid System Library Construction Kit (Stratagene). Detailed steps are as follows. The ORF sequences of *MsrA/B* and candidate genes were linked to the bacterial two-hybrid vectors pBT (bait expression vector) and pTRG (prey expression vector), respectively. All the gene IDs are listed in **Supplementary Table 2**. Then, recombinant pBT and pTRG vectors were co-transformed into the reporter strain *E. coli* XL1 blue MRF’ for the bacterial two-hybrid assays. A single colony of the transformed strain into 5 ml LB liquid medium, and the culture was shaked at 37 °C for about 18 h. 1 ml of bacterial solution were taken and centrifuged at 2500 g for 2min at room temperature. After discarding the supernatant, 1ml M9^+^ His dropout broth was added to clean the bacteria. After cleaning twice, 1ml M9^+^ His was added, gently blowed and absorbed the resuspended bacteria with a pipette to prepare a spot sample bacterial solution. 5 -10 μL of the prepared spot bacterial solution were respectively planted on the corresponding grid positions of DSSM (dual selective screening medium) and NSSM (nonselective screening medium plates), respectively. The plate was inverted in a 37 °C incubator to avoid light. After 12 hours, the plate was taken out to observe and record the colony growth, then continue to culture the plate, and observe and record the colony growth per 8 hours according to the above method until 72 hours. Co-transformed strains containing pBT-LGF2/pTRG-Gal11P and pBT/pTRG were used as the positive and the negative control, respectively. Hem and OsmC are two different antioxidant enzymes and lsrA is a LysR family transcriptional regulator, and they were used as negative control. All the nucleotide sequence reported in this article has been submitted to GenBank and their accession numbers were listed in **Supplementary Table 2**.

### 2.14 Statistical analysis

All data and statistical significance were analyzed using the independent-sample T test, Duncan’s test, and single factor ANOVA method by IBM SPSS Statistics Version 20. The bars indicate the standard deviations (SD) of three independent experiments. *P* values < 0.05 or < 0.01 were considered as statistically significant. All experiments were conducted with at least three biological replicates.

## 3 Results

### 3.1 Characterization and phylogentic analysis of Msrs

Four *msr* genes were identified from the *M. huakuii*7653R genome, including *MCHK_3347*, *MCHK_5276*, *MCHK_5689* and *MCHK_5902*, which was named *msrA1*, *msrB1*, *msrA2* and *msrB2*, respectively. And they encode MsrA1, MsrB1, MsrA2 and MsrB2, respectively. Conserved domain analysis revealed that MsrA1 and MsrA2 belong to the PMSR super family and encode the MsrA, which can catalyze methionine-*S*-sulfoxide (Met-*S*-O) into methionine (Achilli et al., 2015). MsrB1 and MsrB2 belong to the SeIR super family and encode the MsrB, which can catalyze methionine-*R*-sulfoxide (Met-*R*-O) into methionine (Kryukov et al., 2002) (**Supplementary Figure 5**). To investigate the distribution of *Msrs* in different species of rhizobia, two methods were performed. Firstly, the name of gene of Msrs were used to search and compare the genomes of rhizobia from different genera; Secondly, the BLASTP have been carried out with key domains of Msrs as query and other rhizobia protein database as targets. We analyzed the genomic sequences of *M. huakuii* 7653R, *M. japonicum* MAFF 303099, *Bradyrhizobium diazoefficiens* USDA 110, *Sinorhizobium meliloti* 1021, *Rhizobium etli* CFN42, *R. leguminosarum* bv. trifolii WSM2304, *S. fredii* NGR234, *Azorhizobium caulinodans* ORS 571, *S. fredii* HH103 and *B. japonicum* USDA 6. As a result, 25 *MsrAs* and 24 *MsrBs* were identified, whose number and distribution varied among different rhizobial species (**Supplementary Table 3**). Blast conserved sequences of rhizobial species and some typical strains, such as *Escherichia coli*, *Mycoplasma genitalium*, *Mycobacterium smegmatis*, *Xanthomonas campestris*, *Francisella tularensis* and *Staphylococcus aureus*, these blast results showed that MsrAs contained a conserved motif GCFW at the N terminal (**Supplementary Figure 6**), and MsrBs harbored two conserved motifs GWPS and RXCX (**Supplementary Figure 7**). Two pairs of Zn^2+^-conjugating Cys were distributed in MsrBs and the Cys of RXCX was involved in the redox process.

To investigate the evolutionary relationship of MsrAs and MsrBs in different species, phylogenetic trees were constructed with the Neighbor-Joining method of MEGA 6.0 software. The results indicated that MsrA1, MsrA2, MsrB1 and MsrB2 in *M. huakuii* 7653R were the closest to those of *M. japonicum* MAFF 303099, and MsrAs or MsrBs in the same rhizobial genus had closer evolutionary relationships and tended to be clustered in the same branch (**Supplementary Figures 8**, **9**).

### 3.2 Expression characteristics of *msrs* during symbiosis and in response to oxidative stress

**Figure 1:**
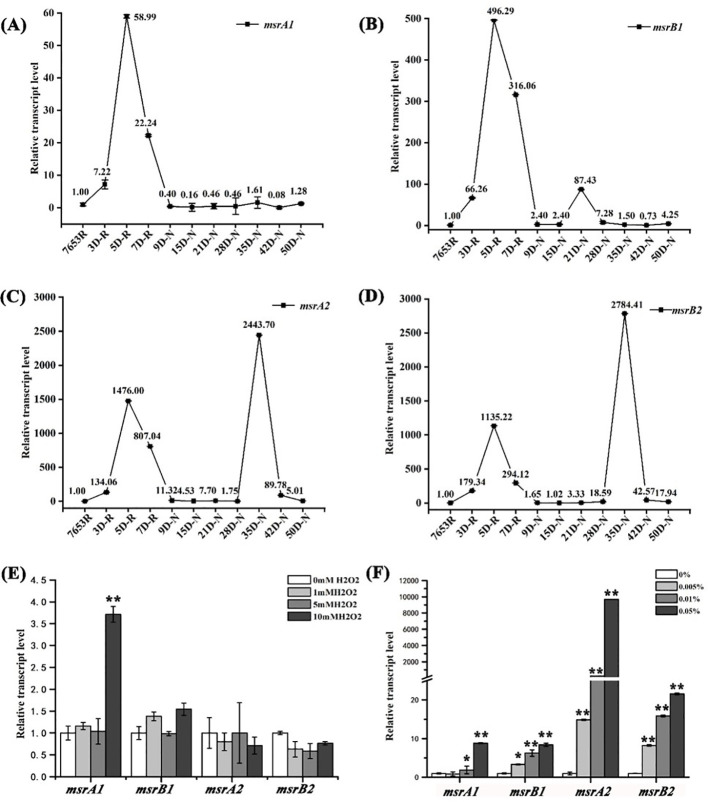
Expression profiles of *msrA* and *msrB* genes during symbiosis and in response to oxidative stress. **(A–D)** Temporal expression patterns of *msrA1*, *msrB1*, *msrA2* and *msrB2* in free-living *M. huakuii* 7653R and in roots (R) or nodules (N) at different days post-inoculation (DPI). **(E, F)** Transcript levels of *msr* genes in response to increasing concentrations of hydrogen peroxide (H_2_O_2_) and sodium hypochlorite (NaClO), respectively. rnpB was used as the internal reference for transcriptional level detection. The transcription level of each gene in free-living wild-type 7653R was set as the reference (1.0), and all values on the Y-axis represent the relative fold change of gene transcription levels in different samples compared with the free-living wild-type 7653R. Data are mean ± SD of three biological replicates. *p < 0.05, **p < 0.01 vs. control (Student’s *t*-test).

To investigate the temporal expression patterns of *msr* genes during symbiosis, transcript levels of *msrs* were analyzed in free-living *M. huakuii* 7653R as well as in roots (R) and nodules (N) at various time points post-inoculation. The expression profile of *msrA1* (**Figure 1A**) showed a sharp peak in roots at 5 days post-inoculation (5D-R; 58.99-fold induction), followed by a rapid decline (7D-R; 22.24-fold) and persistently low expression in later root and nodule stages (e.g., 9D-N to 50D-N; 0.16–1.28-fold). In contrast, the induction of *msrB1* (**Figure 1B**) was much more pronounced. Its expression was upregulated by 66.26-fold in roots at 3 days (3D-R), reaching a peak of 496.29-fold in roots at 5 days (5D-R). Afterwards, its expression decreased sharply to 316.06-fold at 7 days in roots (7D-R). It maintained a relatively high transcriptional level of 87.43-fold in nodules at 21 days, while remaining at relatively low fluctuating levels at all other time points. The *msrA2* gene (**Figure 1C**) displayed two distinct expression peaks: a moderate increase at 3D-R (134.06-fold), rising to 1476.00-fold at 5D-R, followed by a decline (7D-R; 807.04-fold), and a second, stronger peak in nodules at 35 days (35D-N; 2443.70-fold), before decreasing to near-basal levels by 50D-N (5.01-fold). For *msrB2* (**Figure 1D**), transcript levels increased gradually from 179.34-fold (3D-R) to 1135.22-fold (5D-R), then declined (7D-R; 294.12-fold) and remained low until a substantial induction occurred at 35D-N (2784.41-fold), after which expression dropped rapidly to basal levels (50D-N; 17.94-fold).

To determine whether *msr* genes are induced by oxidative stress in rhizobia, induction experiments were performed. *M. huakuii* 7653R were treated with different concentrations of hydrogen peroxide and sodium hypochlorite for 30 minutes, and then the changes of *msr* genes transcription levels were determined. Results showed that the transcription level of *msrA1* was significantly upregulated (approximately 10-fold) upon treatment with 10 mM H_2_O_2_. In contrast, the expression of *msrB1*, *msrA2*, and *msrB2* did not change significantly when *M. huakuii* 7653R was exposed to different concentrations of H_2_O_2_ (**Figure 1E**). Furthermore, treatment with sodium hypochlorite (NaClO) increased the transcript levels of all four genes (*msrA1*, *msrB1*, *msrA2*, and *msrB2*) in a concentration-dependent manner (**Figure 1F**).

### 3.3 Growth curve, motility and biofilm formation capacity of *msrs* overexpression and deletion mutant strains

**Figure 2:**
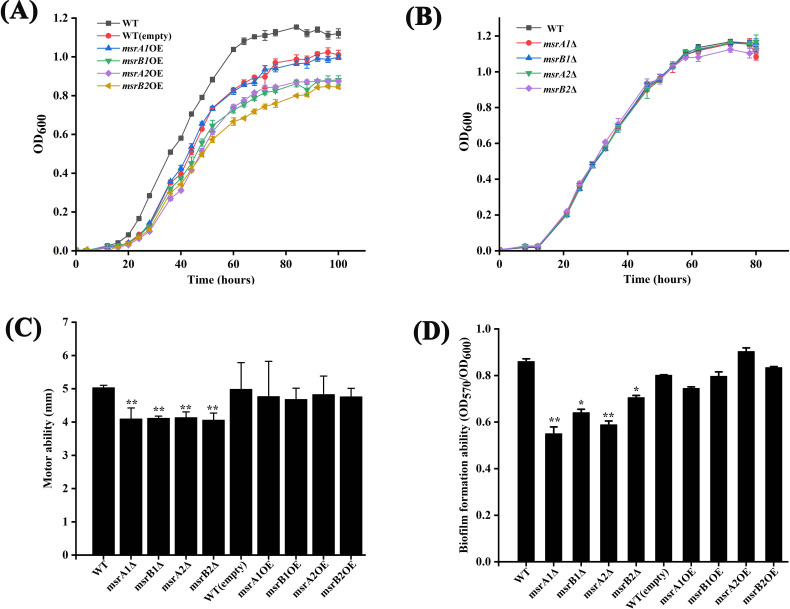
Growth, motility, and biofilm formation of *msr* native promoter and deletion mutant strains. **(A)** Growth curves of wild-type (WT), empty vector control [WT (empty)], and *msr* native promoter strains (*msrA1OE*, *msrB1OE*, *msrA2OE*, *msrB2OE*). **(B)** Growth curves of WT and *msr* deletion mutants (*msrA1Δ*, *msrB1Δ*, *msrA2Δ*, *msrB2Δ*). **(C)** Motility and **(D)** biofilm formation of the indicated strains. WT refers to *M. huakuii* 7653R; WT (empty) indicates WT harboring the empty vector pBBR1MCS-5. Data are presented as mean ± SD of three independent experiments. *P < 0.05, **P < 0.01 (independent-sample t-test).

To investigate the functional roles of *msr* genes in symbiotic nitrogen fixation, overexpression and deletion mutants of each *msr* gene were generated in *M. huakuii* 7653R. The native promoter strains were verified by PCR amplification using universal primers PBBR-F/R, followed by sequencing of the products (**Supplementary Figure 10A**), the deletion mutants (*msrA1Δ*, *msrB1Δ*, *msrA2Δ*, and *msrB2Δ*) were constructed via the *Cre-lox* system and confirmed by PCR with gene-specific primers (**Supplementary Figure 10B**). The plasmid map of pBBR1MCS-5 and representative sequencing results are presented in **Supplementary Figure 11A**. Sequencing of the PCR products verified the complete removal of the respective *msr* open reading frames (**Supplementary Figure 11B**). Total RNA was extracted from the wild-type (WT), *msrA1OE*, *msrB1OE*, *msrA2OE*, and *msrB2OE* strains. Quantitative PCR analysis showed that the relative transcript levels of *msrA1*, *msrB1*, *msrA2*, and *msrB2* were all successfully overexpressed in the native promoter strains and in nodules inoculated these strains, confirming successful overexpression at the transcriptional level (**Supplementary Figures 12A, B**). Growth curve analysis revealed that all native promoter strains and the WT strain carrying the empty vector [WT (empty)], exhibited slower growth rates compared to the WT strain (**Figure 2A**), due to additional metabolic burdens, including energy consumption for plasmid replication, expression of resistance genes, and mild oxidative stress. Among them, *msrA1OE* displayed a growth trend similar to WT (empty), *msrB1OE* and *msrA2OE* showed comparable growth patterns, and *msrB2OE* demonstrated the slowest growth rate. In contrast, the growth rates of all deletion mutants did not differ significantly from that of the WT strain (**Figure 2B**). Motility assays (**Figure 2C**) showed that all deletion mutants (*msrA1Δ*, *msrB1Δ*, *msrA2Δ*, and *msrB2Δ*) exhibited significantly reduced motility (approximately 4.0 mm) compared to the WT (approximately 5.0 mm; *p* < 0.01). However, the motor ability of native promoter strains showed no difference comparable to the WT.

In biofilm formation assays (**Figure 2D**), all deletion mutants, *msrB1*OE and *msrB2* OE strains displayed a marked decrease in biofilm formation (OD_570_/OD_600_ ≈ 0.55–0.70) relative to the WT (OD_570_/OD_600_ ≈ 0.85; *p* < 0.05 or *p* < 0.01). However, *msrA1*OE *and msrA2*OE strains have no significant change compared with WT.

### 3.4 Alterations in oxidative stress resistance and antioxidant enzyme activity in *msr* overexpression and deletion mutants

**Figure 3:**
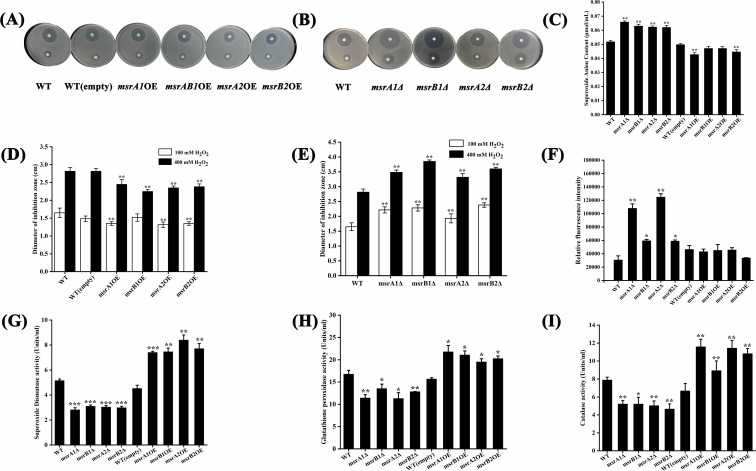
Oxidative stress resistance and antioxidant enzyme activities in different strains. **(A, B)**, represent the inhibition zone images for *msr* native promoter strains and deletion mutants treated with 100 mM and 400 mM H_2_O_2,_ respectively. **(C)**, determination the intracellular superoxide anion content in different strains. **(D, E)**, represent the quantitative analysis of inhibition zone sizes, corresponding to **(A, B)**, respectively. **(F)**, relative fluorescence intensity of H_2_O_2_. **(G–I)**, indicate the total activities of CAT, GPx and SOD in different strains, respectively. WT, *M. huakuii* 7653R; WT (empty), WT carrying the empty overexpression vector. Data are mean ± SD from three independent experiments. *p < 0.05, **p < 0.01 (independent-sample t-test).

To investigate the contribution of Msrs to oxidative stress tolerance, resistance to H_2_O_2_ was assessed using a filter paper disc assay. At concentrations of 100 mM and 400 mM H_2_O_2_, the *msr* native promoter strains exhibited significantly smaller inhibition zones compared to the wild-type *M. huakuii* 7653R and WT (empty) (**Figures 3A, D**). Conversely, the *msr* deletion mutants produced significantly larger inhibition zones than the WT (**Figures 3B, E**).

Intracellular superoxide anion and H_2_O_2_ content were measured in *msr*-OE and deletion mutant strains. The results showed that the superoxide anion content was significantly elevated in all *msr* deletion mutants relative to the wild type, whereas it was significantly reduced only in the *msrA1* and *msrB2* overexpression strains (**Figure 3C**). H_2_O_2_ content were significantly higher in all *msr* deletion mutants, showing 2 to 6-fold increases compared to the wild type, however, no detailed differences in *msr*-OE strains (*p* < 0.01 or *p* < 0.05; **Figure 3F**).

To further explore the mechanism underlying Msr-mediated oxidative stress resistance, the activities of three key antioxidant enzymes — catalase (CAT), glutathione peroxidase (GPx), and superoxide dismutase (SOD) — were measured in different strains. Compared to the wild type, all *msr* deletion mutants (*msrA1Δ*, *msrB1Δ*, *msrA2Δ* and *msrB2Δ*) exhibited significantly reduced activities of SOD (*p* < 0.001, approximately 50% reduction; **Figure 3G**), GPx (*p* < 0.05/*p* < 0.01; **Figure 3H**), and CAT (*p* < 0.05/*p* < 0.01; **Figure 3I**). In contrast, all *msr* native promoter strains (*msrA1OE*, *msrB1OE*, *msrA2OE*, *msrB2OE*) showed elevated enzyme activities: an approximately 2-fold increase for SOD (*p* < 0.05/*p* < 0.01), significant increases for GPx, and a 1.5 – 2.0-fold increase for CAT. The WT (empty) control did not differ significantly from the wild type in any enzyme activity assay.

### 3.5 Transcriptional profiles of antioxidant enzyme genes in *msr* mutants and native promoter strains

To assess the impact of *msr* deletion and overexpression on the transcription of key antioxidant enzyme genes, we performed quantitative real-time polymerase chain reaction (qRT-PCR) analysis. The transcript levels of five genes — encoding superoxide dismutases (*sodA*, *sodB*), catalases (*katE*, *katG*), and a peroxiredoxin (*prx*) — were measured using gene-specific primers (**Supplementary Table 1**), with *rnpb* serving as the internal reference gene for normalization.

**Figure 4:**
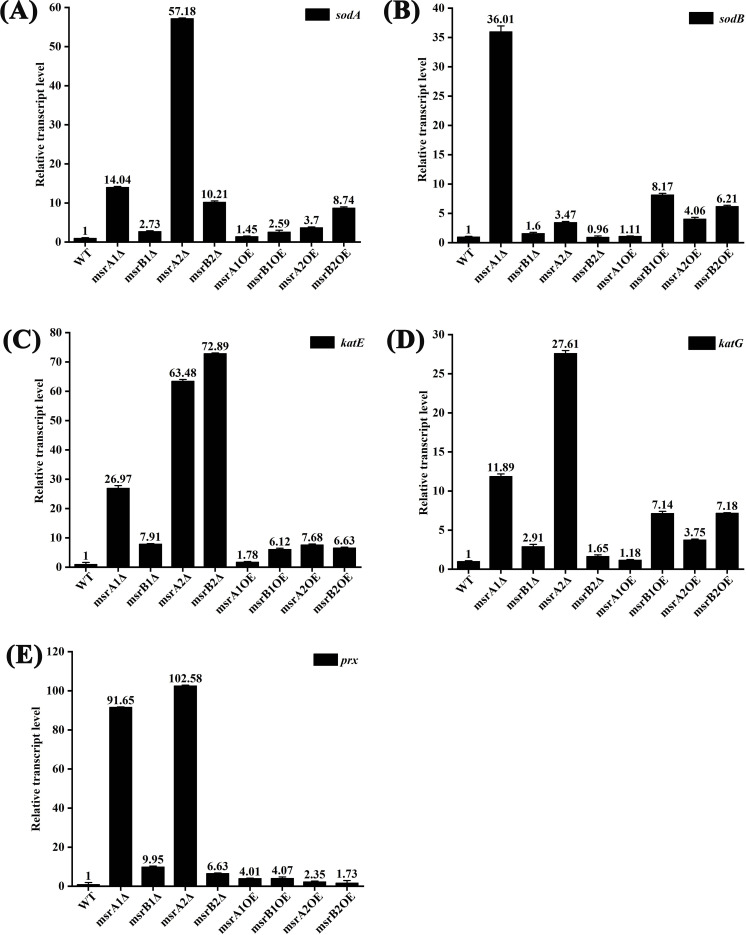
Transcriptional profiles of antioxidant enzyme genes in *msr* mutants and native promoter strains. Transcript levels of **(A)** *sodA*, **(B)** *sodB*, **(C)** *katE*, **(D)** *katG*, and **(E)** *prx* in the indicated strains, normalized to levels in the wild-type (WT). Values in the bars represent fold-change relative to the WT. Gene expression was quantified by qRT-PCR using *rnpB* as the internal reference. Data are presented as mean ± SD of three independent experiments.

As shown in **Figures 4A–E**, the expression patterns of these five antioxidant enzyme genes exhibited distinct changes across the *msr* mutant strains. Four genes (*sodA*, *sodB*, *katG*, and *prx*) displayed the most pronounced upregulation in the *msrA1Δ* and *msrB1Δ* mutants. In contrast, their induction was moderate in *msrA2Δ* and *msrB2Δ* (e.g., *sodA* and *prx*) or remained comparable to wild-type levels (e.g., *sodA* and *sodB* in these strains). The transcript level of *katE* was elevated to varying degrees (7.91- to 72.89-fold) in all four *msr* deletion mutants (**Figure 4C**).

In the *msr* native promoter strains (**Figures 4A–E**), four genes (*sodA*, *sodB*, *katE*, and *katG*) showed transcript levels similar to the wild type only in the *msrA1OE* strain. In the other three native promoter strains (*msrB1OE*, *msrA2OE*, and *msrB2OE*), their expression was significantly upregulated, though generally less than 10-fold (ranging from 2.59- to 8.74-fold). The expression of *prx* remained largely unchanged in *msrB2OE* (1.73-fold vs. wild type) but was moderately increased (2.35- to 4.01-fold) in the remaining native promoter strains (**Figure 4E**).

### 3.6 Effect of *msr* overexpression and deletion on symbiotic phenotype

To assess the impact of *msr* overexpression on symbiosis, *A. sinicus* were inoculated with the native promoter strains *msrA1OE*, *msrB1OE*, *msrA2OE*, and *msrB2OE*. The symbiotic phenotype was examined at 30 days post-inoculation (dpi). qRT-PCR analysis confirmed that all four *msr* genes were overexpressed in the nodules formed by their respective strains compared to those induced by the wild-type *M. huakuii* 7653R (**Supplementary Figure 12C**).

**Figure 5:**
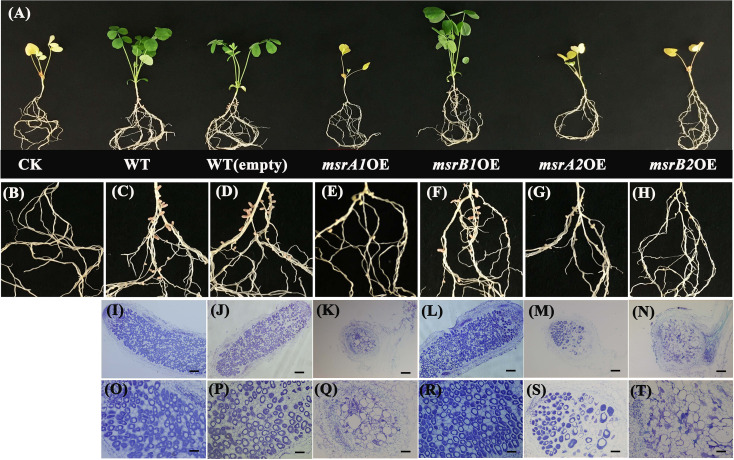
Symbiotic phenotypes and longitudinal paraffin sections induced by *msr* native promoter strains. **(A)**, Whole-plant phenotypes: from left to right, non-inoculated control, wild-type (WT), empty vector control [WT (empty)], *msrA1*OE, *msrB1*OE, *msrA2*OE, and *msrB2*OE. **(B)**, Representative root image of the non-inoculated control. **(C–H)**, Representative images of roots and nodules inoculated with WT **(C)**, WT (empty) **(D)**, *msrA1*OE **(E)**, *msrB1*OE **(F)**, *msrA2*OE **(G)**, and *msrB2*OE **(H)**, respectively. **(I)** and **(J)**, Nodules induced by WT and WT (empty), respectively. **(K–N)**, Nodules induced by the native promoter strains *msrA1*OE, *msrB1*OE, *msrA2*OE, and *msrB2*OE, respectively. **(O–T)**, Magnified views of the nitrogen-fixing zones corresponding to panels **(I–N)**, respectively. WT: *M. huakuii* 7653R; WT (empty): WT harboring the empty vector. All images were taken and nodules were collected at 30dpi. Scale bars: 500 μm **(I–N)**; 200 μm **(O–T)**.

Phenotypic observation revealed that plants inoculated with *msrA1OE*, *msrA2OE*, or *msrB2OE* were stunted, displayed yellow leaves, and developed white nodules (**Figures 5A, E, G, H**). In contrast, plants inoculated with WT (empty) or *msrB1OE* remained green and formed pink nodules (**Figures 5A, D, F**). Relative to wild-type inoculation, inoculation with *msrA1OE*, *msrA2OE*, or *msrB2OE* significantly reduced both the shoot fresh weight and nitrogen fixation activity. Additionally, inoculation with *msrA2OE* and *msrB2OE* significantly decreased nodule number. No significant differences in symbiotic parameters were observed between plants inoculated with WT (empty) and *msrB1OE* (**Table 1**).

**Table 1:** Quantitative analysis of symbiotic phenotype of *Msrs* overexpression.

Strains	Fresh weight of the above ground biomass (g/plant)	Number of nodules (/plant)	Nitrogen fixation activity (μmol/g*h)
CK	0.04 ± 0.01	0	0
WT	0.30 ± 0.07	26.56 ± 9.21	32.85 ± 6.88
WT empty)	0.30 ± 0.07	23.22 ± 5.92	34.89 ± 7.43
*msrA1*OE	0.05 ± 0.01**	19.11 ± 9.16	3.32 ± 1.12**
*msrB1*OE	0.38 ± 0.11	19.22 ± 7.35	30.66 ± 5.31
*msrA2*OE	0.05 ± 0.01**	15.22 ± 4.52**	0.49 ± 0.03**
*msrB2*OE	0.04 ± 0.01**	17.56 ± 5.58**	6.18 ± 3.77**

Symbiotic phenotype was observed at 30 DPI, * * indicated significance difference (*p* < 0.01). Significant differences were determined by Student’s *t* test. The error bars represented the standard deviations of three independent experiments. WT indicated *M. huakuii* 7653R, WT (empty) indicated *M. huakuii* 7653R containing the empty overexpression vector.

**Figure 6:**
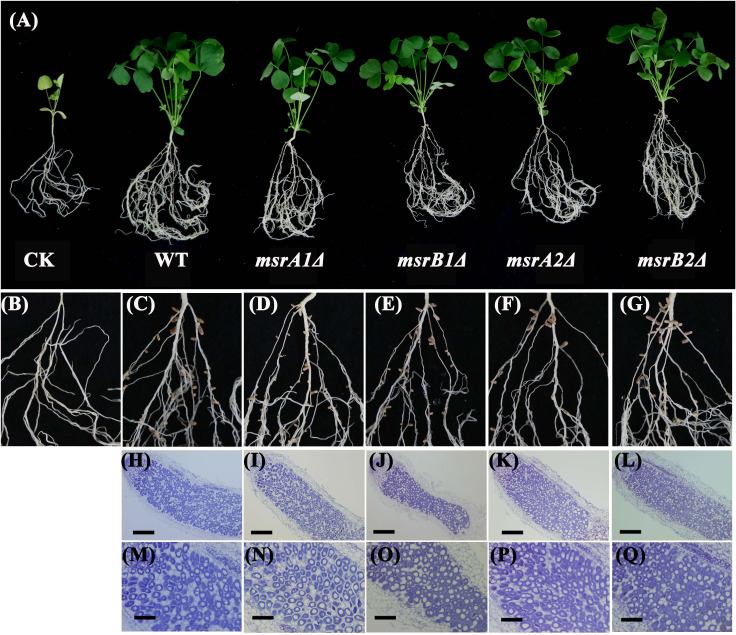
Symbiotic phenotypes and longitudinal paraffin sections induced by *msr* deletion mutants. **(A)**, Whole-plant phenotypes at 33 DPI: from left to right, non-inoculated control, wild-type (WT), *msrA1Δ*, *msrB1Δ*, *msrA2Δ*, and *msrB2Δ*. **(B)**, Representative root system of the non-inoculated control. **(C–G)**, Representative images of roots and nodules from plants inoculated with WT, *msrA1Δ*, *msrB1Δ*, *msrA2Δ*, and *msrB2Δ*. **(H–L)**, Nodules induced by the *msr* deletion mutants wild-type (WT), *msrA1Δ*, *msrB1Δ*, *msrA2Δ*, and *msrB2Δ*, respectively. **(M–Q)**, Magnified views of the nitrogen-fixing zones corresponding to panels **(I–N)**, respectively. All images were taken and nodules were collected at 33dpi. Scale bars: 500 μm **(H–L)**; 200 μm **(M–Q)**.

To examine nodule internal structure, all nodules were collected at 30 dpi, sectioned, and stained with toluidine blue. Microscopic observation showed that nodules induced by *msrA1OE*, *msrA2OE*, and *msrB2OE* were smaller, more spherical, and contained fewer infected plant cells compared to wild-type nodules (**Figures 6K, M, N, Q, S, T**). Nodules formed by *msrB1OE* exhibited no obvious morphological differences from the wild-type control (**Figures 6L, R**).

**Figure 7:**
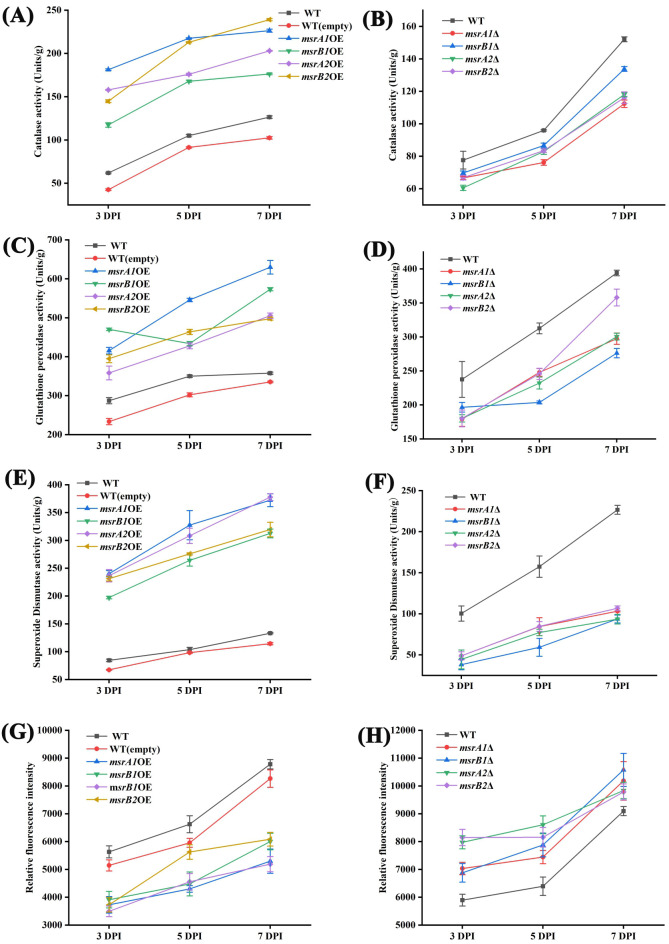
Antioxidant enzyme activity and intracellular hydrogen peroxide in roots of *A. sinicus* inoculated with different strains over time. **(A, C, E)** Activities of catalase (CAT), glutathione peroxidase (GPx), and superoxide dismutase (SOD), respectively, in roots at 3, 5 and 7 DPI with wild-type (WT), empty vector control [WT (empty)], or *msr* native promoter strains. **(B, D, F)** Corresponding activities in roots inoculated with WT or *msr* deletion mutants. **(G)** Relative H_2_O_2_ fluorescence intensity in roots from **(A, C, E)**, measured using the H_2_O_2_-specific fluorescent probe HYTZ-1. **(H)** Relative H_2_O_2_ fluorescence intensity in roots from **(B, D, F)**. WT: *M. huakuii* 7653R. Data are mean ± SD of three independent experiments.

To evaluate the effect of *msr* deletion on symbiosis, the symbiotic phenotype of plants inoculated with the deletion mutants (*msrA1Δ*, *msrB1Δ*, *msrA2Δ*, and *msrB2Δ*) was analyzed at 33 dpi. All plants inoculated with the mutant strains showed vigorous growth and formed red nodules (**Figures 7A–G**). Quantitative analysis indicated that inoculation with *msrA2Δ* significantly reduced both shoot fresh weight and nodule number, although nitrogen fixation activity remained unchanged. Inoculation with *msrB2Δ* significantly decreased nodule number but increased nitrogen fixation activity, with no significant effect on shoot fresh weight (**Table 2**). Plants inoculated with *msrA1Δ* or *msrB1Δ* showed no significant differences from the wild-type control in any measured symbiotic parameter (**Table 2**). Toluidine blue staining and microscopic observation revealed no obvious differences in nodule morphology or infected cell numbers between plants inoculated with the deletion mutants and the wild-type control (**Figures 7H–Q**).

**Table 2:** Quantitative analysis of symbiotic phenotype of deletion mutants.

Strains	Fresh weight of the above ground biomass (g/plant)	Number of nodules (/plant)	Nitrogen fixation activity (µmol/g*h)
CK	0.07 ± 0.01	0	0
WT	0.61 ± 0.14	62.75 ± 23.48	10.81 ± 2.19
*msrA1Δ*	0.60 ± 0.14	49.42 ± 15.55	10.33 ± 0.74
*msrB1Δ*	0.72 ± 0.27	55.33 ± 28.76	10.33 ± 0.29
*msrA2Δ*	0.54 ± 0.18*	37.17 ± 10.91*	11.03 ± 1.25
*msrB2Δ*	0.64 ± 0.17	42.42 ± 10.88*	15.06 ± 3.26*

Symbiotic phenotype was observed at 33 DPI, * indicated significance difference (*p* < 0.05). Significant differences were determined by Student’s *t* test. The error bars represented the standard deviations of three independent experiments. WT indicated *M. huakuii* 7653R.

Meanwhile, we successfully constructed the *msrA1/msrA2* double deletion mutant *msrA1/A2Δ*. Unfortunately, the double-gene mutant of *msrB1/msrB2* was not successfully constructed. After inoculation with *msrA1/A2Δ*, *A. sinicus* exhibited stunted growth and chlorotic leaves, with round and white nodules (**Supplementary Figures 13A, F**). Compared with the wild type, *msrA1/A2Δ* showed significantly reduced shoot fresh weight, nodule number, and nitrogenase activity (**Supplementary Table 4**). These results indicated that *msrA1/A2Δ* severely impaired nodulation and nitrogen fixation. Toluidine blue staining of nodule sections revealed that the nodules of the double mutant contained only a small number of rhizobia, which were mainly restricted to the infection zone. Few rhizobia were detected in the nitrogen-fixing zone and senescent zone (**Supplementary Figures 10J, N**).

### 3.7 Antioxidant enzyme activity and intracellular hydrogen peroxide in roots inoculated with *msr* overexpression and deletion strains at different time points

To evaluate the effect of *msr* overexpression on the antioxidant response of host roots, the activities of CAT, Prx and SOD were measured in roots at 3, 5, and 7 days post-inoculation (DPI) (**Figures 7A, C, E**). In roots inoculated with the four *msr* overexpression (OE) strains, the activities of all three enzymes increased in a time-dependent manner from 3 to 7 DPI. At each time point, roots colonized by the OE strains consistently exhibited higher activities of CAT, Prx, and SOD compared to roots inoculated with the wild-type (WT) strain. Notably, enzyme activities in roots inoculated with WT (empty) were slightly lower than those in WT-inoculated roots. Correspondingly, the relative fluorescence intensity (indicative of H_2_O_2_ levels) was significantly lower in roots inoculated with *msr* OE strains than in the control roots (**Figure 7G**).

To assess the impact of *msr* deletion on host root antioxidant responses, the activities of the same three enzymes were measured in roots inoculated with the deletion mutants at 3, 5, and 7 DPI (**Figures 7B, D, F**). In roots of *A. sinicus* inoculated with any of the four *msr* mutant strains, the activities of CAT, Prx and SOD were consistently lower than those in WT-inoculated roots at all time points. In line with this reduction in antioxidant capacity, the relative fluorescence intensity (reflecting H_2_O_2_ accumulation) was significantly higher in roots inoculated with the *msr* deletion mutants compared to the WT control (**Figure 7H**). These results indicate that deletion of *msr* genes in rhizobia leads to increased oxidative stress and H_2_O_2_ accumulation in inoculated roots.

### 3.8 Transcriptional changes in symbiotic- and defense-related genes in roots inoculated with *msr* overexpression and deletion strains at different time points

To investigate the effects of *msr* gene on host gene expression, the transcript levels of symbiosis-related genes (*AsNIN*, *AsNPL2*) and defense-related genes (*AsFLS2*, *AsPR10*) were analyzed in *A. sinicus* roots inoculated with four *msr*-OE or deletion strains. Root RNA was extracted at 3, 5 and 7 days post-inoculation (dpi) for qRT-PCR analysis.

**Figure 8:**
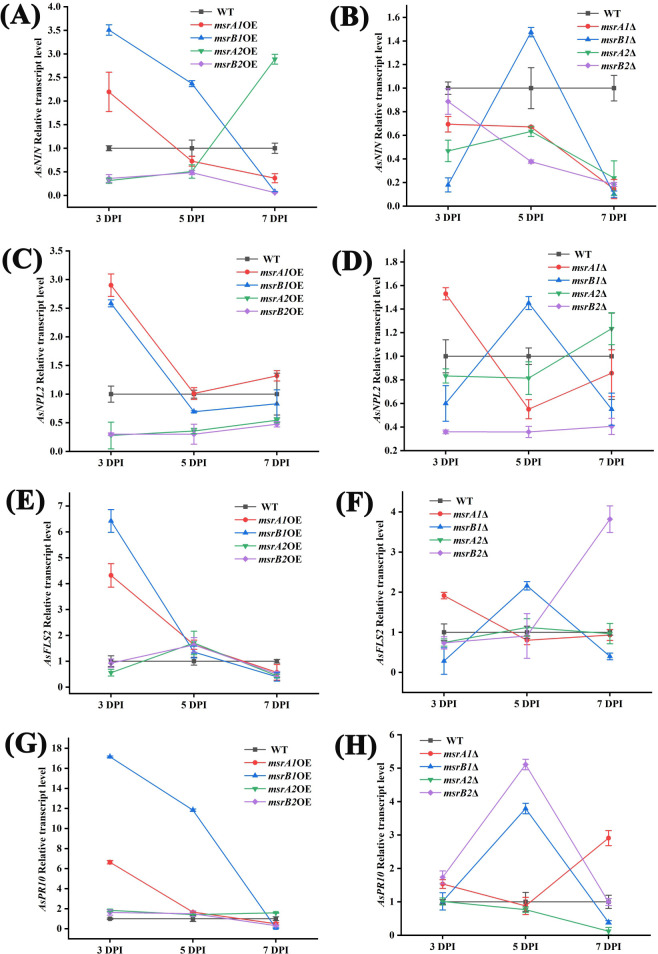
Expression profiles of symbiosis- and defense-related genes in *A. sinicus* roots over time. **(A–D)** Expression levels of the symbiosis-related genes *AsNIN* **(A, B)** and *AsNPL2* **(C, D)** in roots at 3, 5 and 7 DPI with wild-type (WT), *msr* native promoter strains **(A, C)**, or *msr* deletion mutants (Δ) **(B, D)**. **(E–H)** Expression levels of the defense-related genes *AsFLS2* **(E, F)** and *AsPR10* **(G, H)** in roots under the same conditions. WT: *M. huakuii* 7653R. Transcript levels were determined by qRT-PCR and normalized to *AsActin*. Data represent mean ± SD of three independent experiments.

In roots inoculated with *msr* OE strains (**Figures 8A, C**), the expression of symbiosis-related genes *AsNIN* and *AsNPL2* was significantly elevated at 3 DPI in response to the *msrA1OE* and *msrB1OE* strains compared to the wild-type (WT) control, but subsequently declined to WT levels. In contrast, inoculation with *msrA2OE* and *msrB2OE* resulted in lower transcript levels of *AsNIN* (at 3 and 5 DPI) and *AsNPL2* (at 3, 5 and 7 DPI) relative to the WT, except for a transient increase in *AsNIN* at 7 DPI in the *msrA2OE* treatment.

For defense-related genes (**Figures 8E, G**), *AsFLS2* and *AsPR10* expression was also significantly upregulated at 3 DPI in roots inoculated with *msrA1OE* and *msrB1OE*, followed by a gradual decrease to WT levels. The expression of these defense genes in roots inoculated with *msrA2OE* and *msrB2OE* remained similar to the WT control across all time points.

### 3.9 Screening and identification of candidate proteins interacting with MsrA and MsrB proteins

To elucidate the mechanism of *msr* genes in symbiotic nitrogen fixation, candidate interacting proteins for MsrA1, MsrB1, MsrA2, and MsrB2 were screened based on literature review and predictions from the iLoopsServer (http://aleph.upf.edu/iLoopsServer/). The prediction methodology is provided in the “The detailed prediction method of candidate proteins” section of the **Supplementary Data**. The initial candidate lists comprised 1530, 2039, 2276, and 2205 proteins for MsrA1, MsrB1, MsrA2, and MsrB2, respectively (Review-only **Supplementary Materials**). To investigate the antioxidant mechanism, three protein categories were selected for validation: chaperones (GroEL1, GroEL2, GroEL3), antioxidant enzymes (SodA, SodB, KatE, KatG), and a LysR-type regulator (LsrB).

Interactions between these selected proteins and the four Msrs were tested using a bacterial two-hybrid (B_2_H) assay. The genes encoding the target proteins were cloned into the pBT and pTRG vectors, respectively, and co-transformed into the reporter strain *E. coli* XL1-Blue MRF’. Protein interaction was indicated by reporter strain growth on selective DSSM medium.

**Figure 9:**
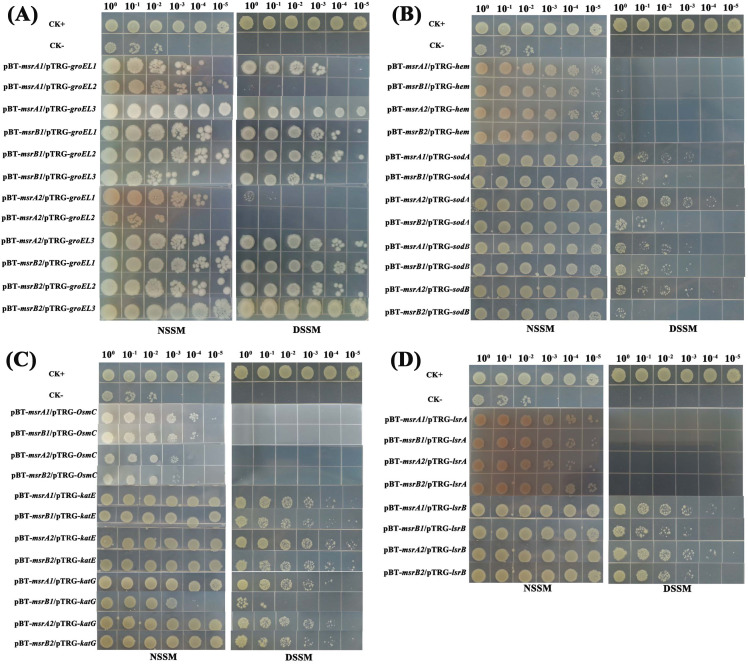
Bacterial two-hybrid assay: interactions between Msrs and substrate proteins. **(A)** GroEL chaperonins. *groEL1, groEL2*, and *groEL3* were cloned into the pTRG vector. **(B)** Superoxide dismutases. *hem* (heme peroxidase, WP_038649367.1), *sodA*, and *sodB* were cloned into the pTRG vector. **(C)** Catalases. *osmC* (OsmC peroxiredoxin, WP_019857426.1), *katE*, and *katG* were cloned into pTRG. **(D)** Transcriptional regulator. *lsrA* (LysR regulator, AID28728.2) and *lsrB* were cloned into pTRG. *msrA1, msrB1, msrA2*, and *msrB2* were individually cloned into the pBT vector. Co-transformants were grown on NSSM and DSSM (5 mM 3-AT + streptomycin). CK+ and CK– are system controls; LsrA served as an interaction-negative control.

The B_2_H results showed that GroEL1 and GroEL3 interacted with all four Msr proteins, whereas GroEL2 interacted only with MsrB1 and MsrB2 (**Figure 9A**). For the antioxidant enzymes, all Msr proteins showed strong interactions with both SodA and SodB, except for a weak interaction observed between MsrB2 and SodB (**Figure 9B**). Similarly, all Msrs strongly interacted with KatE and KatG, with the exception of a weak interaction between MsrB1 and KatG (**Figure 9C**). Two additional antioxidant enzymes, Hem (WP_038649367.1) and OsmC (WP_019857426.1), which showed no interaction with any Msr, served as negative controls (**Figures 9B, C**). Finally, all four Msr proteins were found to interact with the LysR-type regulator LsrB, but not with LsrA (**Figure 9D**).

## 4 Discussion

In the process of rhizobium–legume symbiosis, reactive oxygen species (ROS) play dual roles as critical signal mediators and defensive antimicrobial barriers, limiting rhizobial entry into host plant cells (Pauly et al., 2006; Cardenas et al., 2008; Chang et al., 2009; Nanda et al., 2010; Gourion et al., 2015). Methionine sulfoxide reductases (Msrs) are well recognized as key regulators of oxidative stress tolerance and pathogen defense across different species (Ottemann and Miller, 1997; Ezraty et al., 2005; Sasindran et al., 2007; Lee et al., 2009; Singh et al., 2015; Trivedi et al., 2015; Lee et al., 2017; Martinez et al., 2017; Saha et al., 2017; Singh et al., 2018), However, their systematic functions in symbiotic nitrogen fixation (SNF) remain poorly understood. Here, we comprehensively characterized the sequence features, expression patterns, and molecular mechanisms of four Msr members in *M. huakuii* 7653R during SNF. Combined analyses of physiological phenotypes, biochemical indexes, protein–protein interaction (PPI) profiles, and symbiotic performances enabled us to establish an integrated regulatory model whereby Msrs modulate SNF efficiency. Meanwhile, we also discussed the innovations, limitations, and prospective implications of this work.

Sequence analysis revealed that MsrAs in *M. huakuii* 7653R contain the conserved catalytic motif GCFW, while MsrBs harbor the GWPS/RXCX motif (**Supplementary Figures 6**, **7**), both characteristic of prokaryotic MetO reductases and indicative of their role in methionine sulfoxide repair (Boschi-Muller et al., 2008). The number, distribution, and isoforms of Msrs vary considerably among bacteria, *Staphylococcus aureus* encodes three MsrAs and one MsrB, whereas *Vibrio cholerae* possesses two MsrAs and three MsrBs (Ezraty et al., 2005; Singh et al., 2015; Vanhove et al., 2017; Singh et al., 2018). Such variation likely reflects evolutionary adaptation. The ubiquitous presence of MsrA across eukaryotes, eubacteria, and most archaea underscores its fundamental role in cellular viability (Zhang and Weissbach, 2008). Phylogenetically, the Msrs of *M. huakuii* 7653R cluster most closely with those of other *Mesorhizobium* species, with rhizobial MsrAs and MsrBs forming distinct clades (**Supplementary Figures 8**, **S9**). This phylogenetic pattern correlates with host specificity and symbiotic adaptation, suggesting that Msr evolution may have co-evolved with the rhizobium-legume symbiosis.

Expression profiling further uncovered functional specialization among the four *msrs* paralogs, which is tightly coupled to the dynamic ROS landscape of SNF. All the *msrss* were transiently induced during early infection (peak at 5 DPI in roots), whereas *msrB1*, *msrA2* and *msrB2* exhibited biphasic expression, with peaks at both early infection (5 DPI) and nodule maturation (21 DPI or 35 DPI in nodules), respectively (**Figures 1A–D**). The early ROS burst associated with root hair curling and infection thread formation coincides with the induction of *msrA1*/*msrB1/msrA2/msrB2*, implicating them in initial symbiosis establishment (Saeki, 2011; Damiani et al., 2016; Montiel et al., 2016). At approximately 21–35 DPI, nodules enter the active nitrogen-fixing stage with fully developed and functional bacteroids. Meanwhile, substantial ROS are continuously generated by bacteroid respiration. During this period, the transcription levels of *msrB1*, *msrA2* and *msrB2* reach their second expression peak, which functions to reduce and repair proteins damaged by ROS oxidation. The transcriptional characteristics of *msr* genes demonstrate that different *msr* members perform distinct functions at various stages of symbiotic nitrogen fixation establishment.

Oxidative stress assays further revealed a specialized regulatory pattern: *msrA1* responded specifically to H_2_O_2_, whereas the entire *msr* family was induced under chlorine stress (**Figures 1E, F**). This division of labor enables rhizobia to precisely adapt to the distinct ROS chemistries encountered during symbiosis, a strategy also observed in other bacteria where *msrs* are upregulated under H_2_O_2_ exposure (Singh et al., 2001; Vattanaviboon et al., 2005; Lee et al., 2009). Previous studies have demonstrated that the adaptive response mechanisms of Gram-negative bacteria to hypochlorite stress are closely associated with molecular chaperones (Hsp33, RidA), transcriptional regulators (HypT, RclR, NemR), biofilm formation, and the induction of the viable but non-culturable (VBNC) state (da Cruz Nizer et al., 2020). In rhizobia, the transcription level of *msr* gradually increases with the rising concentration of sodium hypochlorite induction. A possible explanation is that hypochlorite oxidizes certain transcriptional factors in rhizobia, such as HypT, thereby activating the transcription of downstream antioxidant genes, among which the antioxidant enzyme gene *msr* is included.

Physiological and oxidative stress analyses demonstrated that Msrs fine-tune rhizobial fitness to support SNF. Native Msr levels are critical for redox homeostasis, as overexpression impairs bacterial growth while deletion has no significant growth effect (**Figures 2A, B**). In contrast, *msr* deletion significantly reduces rhizobial motility and biofilm formation—two key traits for root attachment and rhizosphere migration (**Figures 2C, D**), highlighting their role in early symbiosis establishment (Hassouni et al., 1999; Beloin et al., 2004; Xiaolin et al., 2020). Msrs also serve as pivotal regulators of rhizobial antioxidant capacity. Overexpression enhances oxidative stress resistance, while deletion increases sensitivity (**Figures 3A, B, D, E**), by upregulating the activity of CAT, GPx, and SOD (**Figures 3G–I**), and modulating the transcription of *sodA/B*, *katE/G*, and *prx* (**Figure 4**). A notable paradox—upregulated antioxidant gene transcription but reduced enzyme activity in Msr mutants—suggests Msrs directly repair oxidatively damaged antioxidant enzymes to preserve function, with native promoter acting as a compensatory response to elevated ROS. This aligns with emerging evidence that rhizobia maintain antioxidant enzyme activity via post-translational modification, positioning Msrs as central mediators of oxidative stress resistance for symbiotic establishment. This model aligns with recent proposals that rhizobia maintain antioxidant enzyme activity via post-translational modification (Wuyu et al., 2024; Weiqin et al., 2025), and underscores the central role of Msrs in mediating oxidative stress resistance to facilitate symbiotic establishment (Lourenco Dos Santos et al., 2018; Kalemba and Stolarska, 2019; Vincent and Ezraty, 2022).

Symbiotic phenotypic and host response analyses further confirmed that finely tuned Msr expression is essential for SNF efficiency. Overexpression of *msrA1*, *msrA2* and *msrB2* induces severe host chlorosis, reduced nitrogenase activity, and diminished bacteroid colonization (**Figure 5**, **Table 1**), while deletion of *msrA2* or *msrB2* (but not *msrA1* or *msrB1*) significantly alters nodulation—with *msrB2* deletion paradoxically increasing nitrogen fixation activity (**Figure 6**, **Table 2**). After inoculation with *msrA1/B2Δ*, *A. sinicus* plants exhibited stunted growth and chlorotic leaves, with round and white nodules (**Supplementary Figure 13**). The weak phenotypic changes of single-gene deletion mutants are caused by gene functional complementation. Rhizobial Msrs also indirectly shape host antioxidant responses: *msr* overexpression increases host root antioxidant enzyme activity and reduces H_2_O_2_ accumulation (**Figures 7**, **8**), while deletion has the opposite effect—consistent with ROS as key symbiotic signals. Gene expression analyses revealed that early *msrA1/msrB1* overexpression upregulates both symbiotic genes (*AsNIN*, *AsNPL2*) and defense-related genes (*AsFLS2*, *AsPR10*), suggesting transient defense gene induction helps attenuate host immune surveillance, while subsequent downregulation facilitates stable symbiosis (**Figures 7**, **8**). In contrast, *msrA2 or msrB2* overexpression suppresses symbiotic gene expression, likely due to excessive ROS scavenging that disrupts symbiotic signaling. The regulation of redox balance is critical for growth, development, and differentiation in living organisms, and multiple genes are known to participate in redox homeostasis during symbiotic nitrogen fixation (Ribeiro et al., 2015). It was reported that the deletion or overexpression of genes encoding superoxide dismutase or peroxidase in rhizobia can alter symbiotic phenotypes (Santos et al., 2000; Jamet et al., 2007; Hanyu et al., 2009; Saeki, 2011). These results indicate that rhizobia can fine-tune symbiotic ROS dynamics through their intrinsic Msr activity, thereby indirectly shaping the host’s antioxidant response. This aligns with the established role of ROS as key symbiotic signals (Damiani et al., 2016; Samuel et al., 2022; Eliane et al., 2025).

Protein interaction networks uncovered the molecular mechanisms underlying Msr function in SNF. Msrs interact with GroELs (**Figure 9A**), forming a conserved prokaryotic mechanism to maintain protein homeostasis—consistent with findings in *Helicobacter pylori (*Melkani et al., 2004; Mahawar et al., 2011). Msrs also bind to antioxidant enzymes SodA, SodB, KatE, KatG (**Figures 9B, C**), establishing a “MetO reduction–ROS scavenging” feedback loop to enhance antioxidant capacity by preserving enzyme activity. This is consistent with previous reports that Msrs can restore the function of oxidatively damaged antioxidant enzymes (Benoit et al., 2013; Lee et al., 2014; Kwak et al., 2016; Sarkhel et al., 2017; Lourenco Dos Santos et al., 2018). Additionally, interaction with LsrB (a LysR-family transcriptional regulator that activates antioxidant genes) (**Figure 9D**), a LysR-family transcriptional regulator known to activate antioxidant genes (Lu et al., 2013; Tang et al., 2014; Tang et al., 2017), forms a “transcriptional regulation–enzyme activity maintenance” cascade, modulating downstream antioxidant gene expression. Such a role is supported by studies showing that Msrs can regulate gene expression by repairing oxidized transcription factors (Drazic et al., 2013; Jiang et al., 2020; Min et al., 2023). In *Sinorhizobium meliloti*, LsrB activates the *lrp3–lpsCDE* operon involved in lipopolysaccharide synthesis and directly induces *gshA* (Jamet et al., 2005; Lu et al., 2013; Tang et al., 2014), which is required for glutathione synthesis and ROS resistance in bacteroids (Frendo et al., 2005). Collectively, these interactions integrate protein repair, ROS scavenging, and transcriptional regulation, enabling Msrs to precisely control oxidative stress during symbiosis.

This study highlights the first systematic characterization of four Msr homologs in *M. huakuii* 7653R and their collective functions in symbiotic nitrogen fixation. We uncovered functional differentiation among Msr paralogs and proposed a conserved “Msr–antioxidant enzyme–host gene” regulatory axis, which deepens our mechanistic understanding of redox homeostasis underlying rhizobium–legume symbiosis. Distinct from Msrs in pathogenic bacteria that mainly govern virulence, rhizobial Msrs coordinate intrinsic antioxidant defense and host symbiotic signaling, reflecting adaptive divergence driven by mutualistic rather than parasitic lifestyles. Furthermore, the screening of Msr-interacting proteins offers promising molecular targets for the future genetic modification of rhizobial strains to improve antioxidant adaptability and nitrogen fixation performance.

Several limitations of the present study warrant further investigation. First, regarding model specificity, cross-system validation in additional classical symbiotic pairs, such as *S. meliloti*–*Medicago truncatula* and *Bradyrhizobium japonicum*–soybean, is essential to verify the conserved roles of Msrs in symbiotic nitrogen fixation. Second, in terms of mechanistic depth, in-depth verification of Msr-interacting proteins and comprehensive exploration of Msr-orchestrated redox regulation in both rhizobia and host plants are required to fully decode the intricate symbiotic regulatory network. Third, considering genetic redundancy, the generation of double or multiple *msrB1/B2* mutant lines using the CRISPR/Cas9 system will help clarify the functional redundancy and compensatory mechanisms among Msr family members. Fourth, for translational application, the rational design of Msr-modified rhizobial strains and subsequent field trials will facilitate the transition from fundamental findings to practical agricultural utilization.

Collectively, these findings demonstrate that the Msr family of *M. huakuii* 7653R possesses conserved structural characteristics and exhibits temporally distinct expression patterns, functioning as central redox regulators during SNF. By modulating antioxidant metabolism, cell motility and biofilm formation, Msrs enhance rhizobial physiological adaptability and assemble functional protein interaction networks. Moreover, Msrs execute cross-kingdom regulatory effects on host antioxidant and symbiotic gene expression, which maintains redox homeostasis, guarantees orderly nodule development, and stabilizes nitrogen fixation efficiency. This work unravels the multilayered regulatory machinery of rhizobial Msrs in microbe–host symbiosis, advances the current knowledge of redox-governed symbiotic coordination, and provides valuable theoretical basis and promising molecular targets for the genetic optimization of rhizobial strains and the sustainable improvement of agricultural nitrogen fixation.

## Data Availability

The datasets presented in this study can be found in online repositories. The names of the repository/repositories and accession number(s) can be found below: https://www.ncbi.nlm.nih.gov/, MCHK_3347 https://www.ncbi.nlm.nih.gov/, MCHK_5276 https://www.ncbi.nlm.nih.gov/, MCHK_5689 https://www.ncbi.nlm.nih.gov/, MCHK_5902 https://www.ncbi.nlm.nih.gov/, MCHK_1528 https://www.ncbi.nlm.nih.gov/, MCHK_2607 https://www.ncbi.nlm.nih.gov/, MCHK_3733 https://www.ncbi.nlm.nih.gov/, MCHK_8063 https://www.ncbi.nlm.nih.gov/, MCHK_1101 https://www.ncbi.nlm.nih.gov/, MCHK_RS21000 https://www.ncbi.nlm.nih.gov/, MCHK_3618 https://www.ncbi.nlm.nih.gov/, MCHK_0509 https://www.ncbi.nlm.nih.gov/, MCHK_RS07210 https://www.ncbi.nlm.nih.gov/, MCHK_0897 https://www.ncbi.nlm.nih.gov/, MCHK_4156 https://www.ncbi.nlm.nih.gov/, MCHK_09420.
