# Supplementary material for: An integrated Msr-antioxidase-host gene circuit maintains redox homeostasis in legume-rhizobium symbiosis
Source: Front Plant Sci. 2026 Jun 3;17:1811549. doi: 10.3389/fpls.2026.1811549 (PMC13272141; doi:10.3389/fpls.2026.1811549)
Supplement: Supplementary file 1 [file DataSheet1.pdf]

Table S1 Primers used in this study

Primers	Sequences (5'-3')	length
**3347OE-F**	GGAATTC CTAA CTCAATGCCTATGACAAGCCGT	978 bp
**3347OE-R**	CGGGATCC TTAGGATGCGGCCTTTTCG	978 bp
**5276 OE-F**	GGAATTC CTAA CTATCTCCACGCCGACGACCG	906 bp
**5276 OE-R**	CGGGATCC TCAGGCCGCCGGCTCG	906 bp
**5689 OE-F**	GGAATTC CTAA ACCAAGTACCTGACCCTGC	1195 bp
**5689 OE-R**	CGGGATCC TCAATTGCTGGCCGACAGA	1195 bp
**5902 OE-F**	GGAATTC CTAA GCTGCTCAAGCCCGAGAACAA	996 bp
**5902 OE-R**	CGGGATCC TCAGGCCGTGGCCG	996 bp
**Universal primer-F**	TGTGGAATTGTGAGCGGATAA	553 bp
**Universal primer-R**	GCGTAGCACCAGGCGTTT	553 bp
**3347 up-F**	GGAATTCTGGACGCCGTCTGCTGG	582 bp
**3347 up-R**	GGGGTACC GTCACGTCTCCTCAAAAGGCA	582 bp
**3347 down-F**	CGGGCCC TGGCACAAAGCATCGTTGG	547 bp
**3347 down-R**	CGAGCTC CGTTTTTTCAGCGCACCG	547 bp
**5276 up-F**	GGAATTCCCATTTCGCACTGCCACTTCT	556 bp
**5276 up-R**	GGGGTACCGCCTTGGCGGCTCGTTCTT	556 bp
**5276 down-F**	CGGGCCC AGGGCATTGCGCAGGCTT	601 bp
**5276 down-R**	CGAGCTC CGCAACGCAAAGCTGGTCC	601 bp
**5689 up-F**	GGAATTCAACGCATCACCCGGCCC	558 bp
**5689 up-R**	GGGGTACCCCGCGGGTAAAGAACGGC	558 bp
**5689 down-F**	CGGGCCC TTACCTCCAAGGGTGGATACG	519 bp
**5689 down-R**	CGAGCTC GCACCTCATCAACCTGTCGAT	519 bp
**5902 up-F**	GGAATTCGGTCCGTTCACCGTCTTCG	560 bp
**5902 up-R**	GGGGTACC GACCATTCCTCCGGTTGTTCC	560 bp
**5902 down-F**	CGGGCCC CGAAATTCGACCGGAAACG	555 bp
**5902 down-R**	ACCGGTCCGACCGGCTGAAGAACG	555 bp
**3347 map-F**	TTCCTCTGCATCCTCACCAT	1755 bp
**3347 map-R**	GTTAAAGCACCATTCGAACG	1755 bp
**5276 map-F**	GGCATAGGCTTGTGATGAGGGA	1912 bp
**5276 map-R**	GAACGGCATCCGGAGGAA	1912 bp
**5689 map-F**	CGCCACCAACTGATTTTCTT	1880 bp
**5689 map-R**	AATCAACAAACCAGGAGAGAA	1880 bp
**5902 map-F**	CCGCCTATGCCGAAAACC	1805 bp
**5902 map-R**	ATGGTGCCGATCAACAACAT	1805 bp
**q3347-F**	AGTGCTTGCCGGTGGCT	112 bp
**q3347-R**	ATAGGTGGCGTTGGCTACATC	112 bp
**q5276-F**	CGGAGCAATATGCGGTCAT	100 bp
**q5276-R**	CAGCCGACGCAGGAAAAT	100 bp
**q5689-F**	CGTCAAGGGCGTCAGCAAG	176 bp
**q5689-R**	TGTGGGCGACCGAGAAATAGA	176 bp
**q5902-F**	GAGTGGAAAGCCATCCTGTCG	105 bp

Table S1 continue.

Primers	Sequences (5'-3')	length
**q5902-R**	GATACCCTTGCGGTGCTCGTT	105 bp
**AsPR10-qF**	ATGCTTTTCTGCTTCTGATCAATGG	127 bp
**AsPR10-qR**	ACACGCCACACAGAAATCTCAAACT	127 bp
**AsFLS2-qF**	CTGGTTATGGTTCTCCCATTGTC	165 bp
**AsFLS2-qR**	CTGCTGTTGAGGAAAGGGTATCG	165 bp
**AsNIN-qF**	GATGCTGATGTGGAGGAGTGTAA	153 bp
**AsNIN-qR**	GCTATGACTATGATGGGATGGAA	153 bp
**Asactin-qF**	GTTCTTTTCCAGCCTTCTATGA	136 bp
**Asactin-qR**	ATGTTTCCGTACAGATCCTTTC	136 bp
**sodA-qF**	TCGCAGCCACAACCCGC	177 bp
**sodA-qR**	TTATTGACGTAGGCCGCATGATG	177 bp
**sodB-qF**	GTCGAAAGAGACGCTGGAATAT	182 bp
**sodB-qR**	ATGGATGTGATTGTAGTGCTGCG	182 bp
**katE-qF**	CAACCGTGCTTGAGGATTTC	149 bp
**katE-qR**	GTCGGCGCGGGTGTATTTAG	149 bp
**katG-qF**	CGAACACCGATGAAAAAAGC	131 bp
**katG-qR**	CGGATCAGACAGGGATGACTG	131 bp
**Prx-qF**	AACAGGCAAGTCCAGCCAATA	137 bp
**Prx-qR**	CCTCATAGCCGGGGTGAAT	137 bp
**Primer RV-M -F**	GAGCGGATAACAATTTCACACAGG	150 bp
**Primer M13-47-R**	CGCCAGGGTTTTCCCAGTCACGAC	150 bp
**RNPB-F**	GGTTTACCGTGCCGCTCCTGTTG	107 bp
**RNPB-R**	GGGAAAGTGCCACAGAAAGCAAACC	107 bp
**pBT-F**	TCCGTTGTGGGGAAAGTTATC	146 bp
**pBT-R**	GGGTAGCCAGCAGCATCC	146 bp
**pTRG-F**	TGGCTGAACAACTGGAAGCT	207 bp
**pTRG-R**	ATTCGTCGCCCGCCATAA	207 bp
**pTRG-sodA-F**	CGGGATCCATGACCGCACTGACTCGCC	747 bp
**pTRG-sodA-R**	CGGAATTCTCAGATCGTCAGGGTGCCCT	747 bp
**pTRG-sodB-F**	CGGGATCCATGGCTTTTGAATTGCCCG	600 bp
**pTRG-sodB-R**	CGGAATTCTCACTTCGCCTTTTCGTAAAGTT	600 bp
**pTRG-Hem-F**	CGGGATCC ATGAACGCTCATCGCGGCA	1740 bp
**pTRG-Hem-R**	CGGAATTC TTAAGCTCCAGGAGTTGTAGGC	1740 bp
**pTRG-katE-F**	CGGGATCCATGGCGAAGCAAGCATCCA	2109 bp
**pTRG-katE-R**	CGGAATTCTCAACCCAGCTTCACCTTCG	2109 bp
**pTRG-katG-F**	CCGCTCGAGATGGACGCGAACACCGATG	2199 bp
**pTRG-katG-R**	GGACTAGTTCAGCCGGCGATGTCGAA	2199 bp
**pTRG-OsmC-F**	CGGGATCCATGACAATTCGCGAAGCCTC	444 bp
**pTRG-OsmC-R**	CCGCTCGAGTTACTGTGCCGTGGTCGC	444 bp
**pTRG-lsrB-F**	CGGGATCCATGGCGTTGGACTGGGACA	600 bp
**pTRG-lsrB-R**	CGGAATTCCTAGAATGACCAACTGCGCG	600 bp
**pTRG-IsrA -F**	CGGGATCCATGGCCATGCTCAACCGCGTTCATC	915 bp

**pTRG-IsrA -R**	CGGAATTCTTAAGCAGCGCTTTCGAGTGAT	915 bp
**PBBR-F**	TGTGGAATTGTGAGCGGATAA	553 bp
**PBBR-R**	GCGTAGCACCAGGCGTTT	553 bp

The underlines indicated the Restriction endonuclease sequences.

Table S2 Gene IDs and protein IDs used in this study

Gene name	Gene ID	Protein ID
msrA1	MCHK_3347	AID31150.1
msrB1	MCHK_5276	AID33070.1
msrA2	MCHK_5689	AID33482.1
msrB2	MCHK_5902	AID33695.1
groEL1	MCHK_1528	WP_038646029.1
groEL2	MCHK_2607	WP_038647117.1
groEL3	MCHK_3733	WP_038648449.1
sodA	MCHK_8063	WP_038654867.1
sodB	MCHK_1101	WP_019857106.1
hem	MCHK_RS21000	WP_038649367.1
katE	MCHK_3618	WP_038648251.1
katG	MCHK_0509	WP_038644931.1
osmC	MCHK_RS07210	WP_019857426.1
lsrA	MCHK_0897	AID28728.2
lsrB	MCHK_4156	WP_010911008.1
rnpb	MCHK_09420	RNase P RNA component class A

Table S3 Location and copy number of MsrAs and MsrBs in different rhizobial strains

Species	Gens in chromosome	Genes in plasmid	*MsrA*	*MsrB*
Mesorhizobium huakuii 7653R	*MCHK_3347 MCHK_5276*<br>*MCHK_5689 MCHK_5902*		**2**	**2**
Mesorhizobium japonicum MAFF 303099	*mll1760 mll4530 mlr3964 mll4820*		**2**	**2**
Bradyrhizobium diazoefficiens USDA 110	*AAV28_28855 blr0834 bll6260 blr7043*<br>*blr7044 bll5855 AAV28_26810*		**4**	**3**
Sinorhizobium meliloti 1021	*SMc02885 SMc01724*<br>*SMc00117 SMc02467*	*SMa1896 SMa1894*	**3**	**3**
Rhizobium etli CFN42	*RHE_CH01247*	*RHE_PB00110 RHE_PE00011*	**2**	**1**
Rhizobium leguminosarum bv. trifolii WSM2304	*Rleg2_0872 Rleg2_3504*	*Rleg2_6004 Rleg2_6581*	**2**	**2**
Sinorhizobium fredii NGR234	*NGR_c07490 NGR_c31330*<br>*NGR_c35410 NGR_c00780*	*NGR_b05680 NGR_b05690*<br>*NGR_b07820*	**3**	**4**
Azorhizobium caulinodans ORS 571	*AZC_0674 AZC_1085*		**1**	**1**
Sinorhizobium fredii HH103	*SFHH103_00079 SFHH103_00687*<br>*SFHH103_03165 SFHH103_03715*	*SFHH103_06366 FHH103_05927*<br>*SFHH103_05928*	**3**	**4**
Bradyrhizobium japonicum USDA 6	*BJ6T_08390 BJ6T_23660 BJ6T_23670*<br>*BJ6T_34380 BJ6T_38520*		**3**	**2**

Data obtained from NCBI database. The red gene number indicated the *MsrA*, the black gene number indicated the *MsrB*.

Table S4 Quantitative analysis of the deletion mutants' symbiotic phenotype.

**Strains**	**Fresh weight of the overground(g/plant)**	**Number of nodule(/plant)**	**Nitrogen fixation activity((μmol/g*h)**
CK	0.04 ± 0.01	0	0
WT	0.18 ± 0.04	23.75 ± 8.02	11.13 ± 2.47
msrA1Δ	0.16 ± 0.03	23.58 ± 4.78	14.04 ± 3.87
msrA2Δ	0.15 ± 0.03*	20.33 ± 4.92	15.10 ± 3.46
msrA1/A2Δ	0.04 ± 0.01**	16.25 ± 6.72*	4.26 ± 1.74**

The symbiotic phenotype was observed at 27 DPI, * indicated significance difference ($p < 0.05$). **indicated very significance difference ($p < 0.01$). Significant differences were determined by Student's t test.

Fig. S1

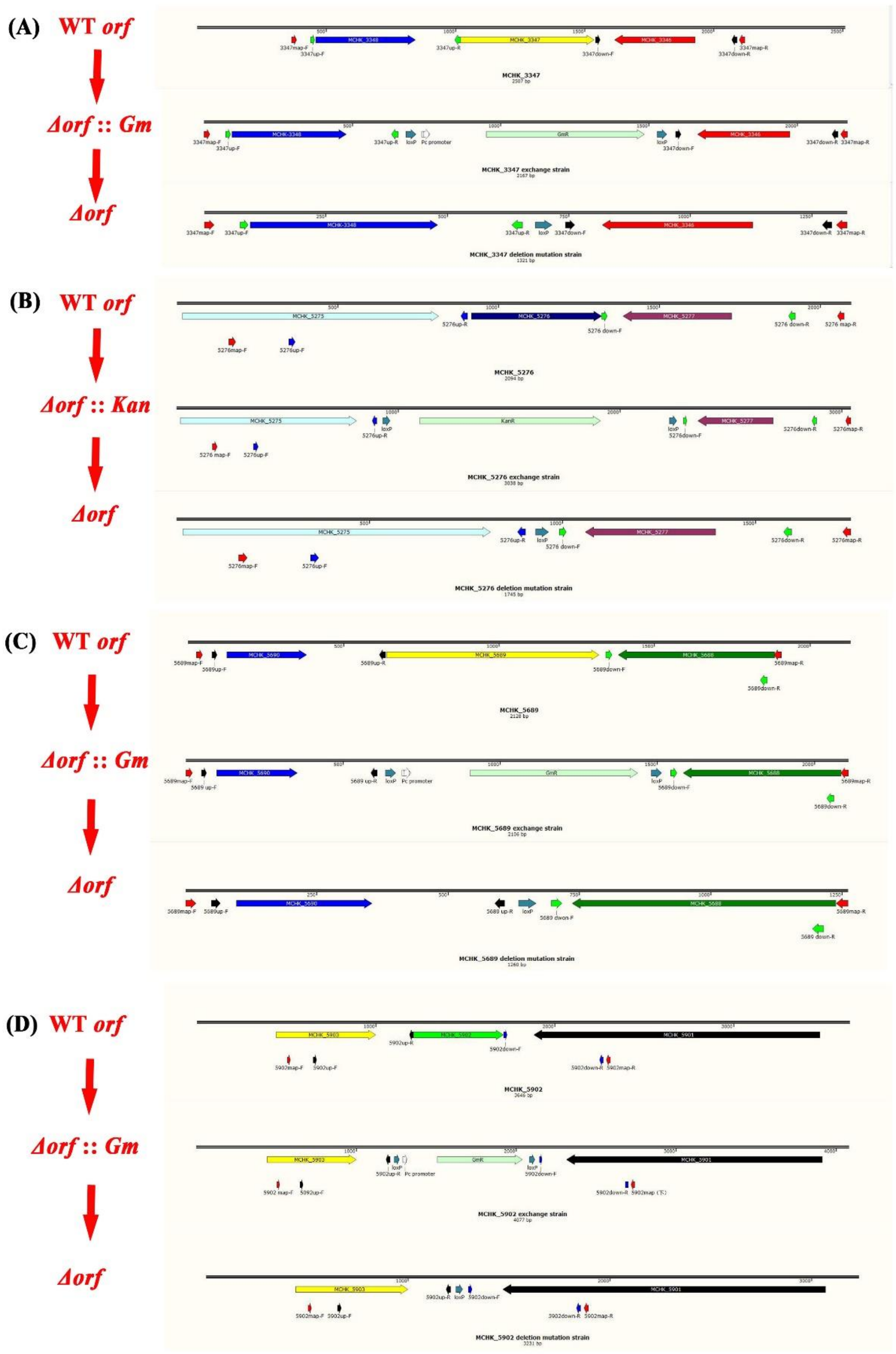
(A)
WT orf
Δorf :: Gm
Δorf
MCHK_3347
MCHK_3347 exchange strain
MCHK_3347 deletion mutation strain
(B)
WT orf
Δorf :: Kan
Δorf
MCHK_5276
MCHK_5276 exchange strain
MCHK_5276 deletion mutation strain
(C)
WT orf
Δorf :: Gm
Δorf
MCHK_5689
MCHK_5689 exchange strain
MCHK_5689 deletion mutation strain
(D)
WT orf
Δorf :: Gm
Δorf
MCHK_5902
MCHK_5902 exchange strain
MCHK_5902 deletion mutation strain

(E) *Cre-lox* system to construct the schematic diagram of deletion mutants (Marx CJ and ME 2002).

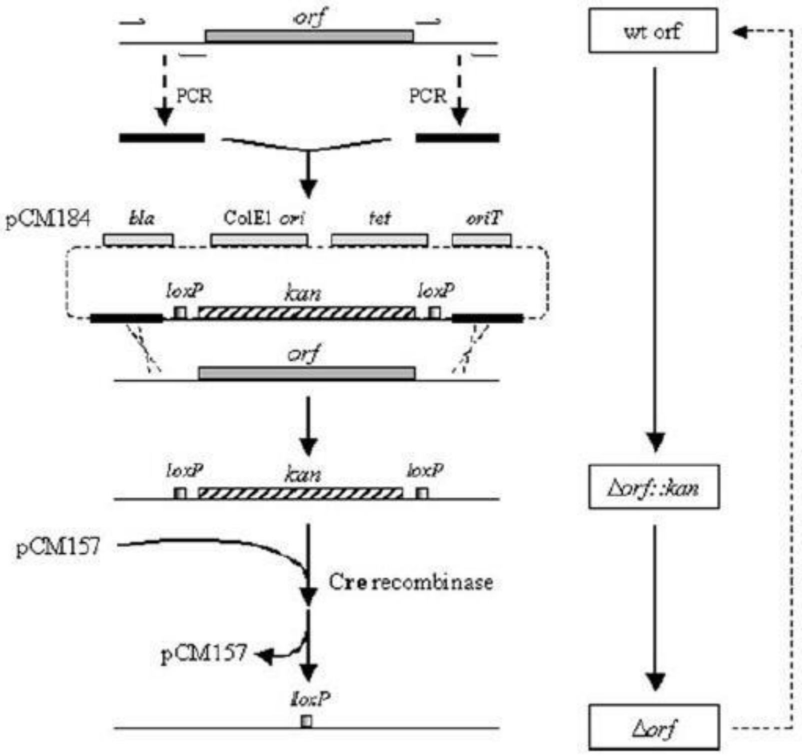

Fig. S1 The construction process of deletion mutant strains. A – D, indicate the construction of *msrA1Δ*, *msrB1Δ*, *msrA2Δ* and *msrB2Δ*, respectively. WT *orf* indicate the position of the deletion gene and its neighbor on the WT chromosome. *Δorf*:: *Kan* (*Gm*) indicate the deletion gene was replaced by the antibiotic gene. *Δorf* indicate the deletion gene was completely absent from the genome. E, *Cre-lox* system to construct the schematic diagram of deletion mutants. Allelic exchange leads to kan (Gm) insertion mutant, which can then be unmarked through the introduction of the cre expression plasmid pCM157 or pCM158. pCM351 was used to construct *msrA1Δ*, *msrA2Δ* and *msrB2Δ*, and pCM158 express Cre recombinase to delete the Gm antibiotic gene. pCM184 was used to construct *msrB1Δ*, and pCM157 express Cre recombinase to delete the Kan antibiotic gene.

The detailed prediction method of candidate proteins

All proteins (about 6325) in *M. huakui* 7653R genome (Bio Sample SAMN 02904634) were predicted for potential 4 Msrs at the protein interaction prediction website (http://sbi.imim.es/iLoopsServer/index.php/init.html). The thoroughly prediction steps were descripted on the supplymentary data. Finally, interacting proteins were obtained and the results were listed in the Review-only supplementary materials.

Firstly, input box (SEQUENCES BOX): sequences of the protein pairs to be tested, in FASTA format. The first word in the FASTA header (between ">" sign and the first whitespace) will be used as identifier code for the protein.

Secondly, input box (INTERACTIONS BOX): the protein pairs to be tested, indicated by the protein identifiers used in the previous box and separated by a double colon "::", and click the button "Submit", as it is shown in Fig. S8 .

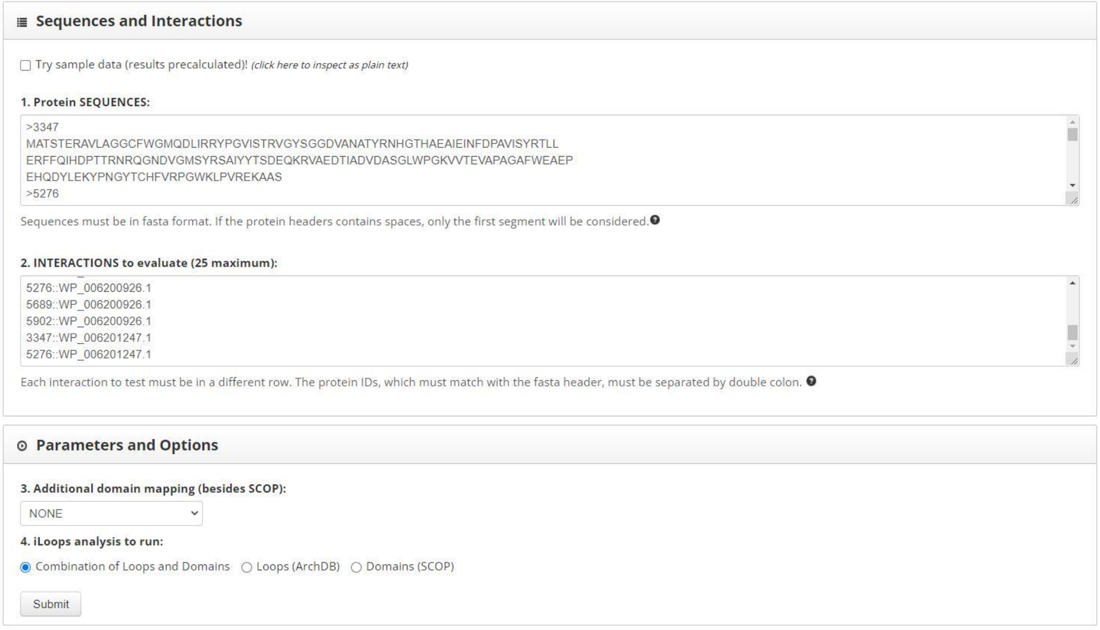

Fig. S2 process of Web site prediction interaction protein

Then, the prompt of successful submission of website prediction interaction protein will appear, after we click Job iD (in Fig. S3), the interaction prediction results will appear in Fig. S4. The results show "YES" indicating that there is interaction between the two proteins, and "N/A" indicating that there is no interaction between the two proteins. According to the above prediction method, the proteins in *M. huakui* 7653R were predicted with 4 Msrs, respectively, and a total of 2620 interacting proteins

were obtained (Table S2). We representatively selected several chaperones, antioxidant enzymes and transcription factors for interaction verification.

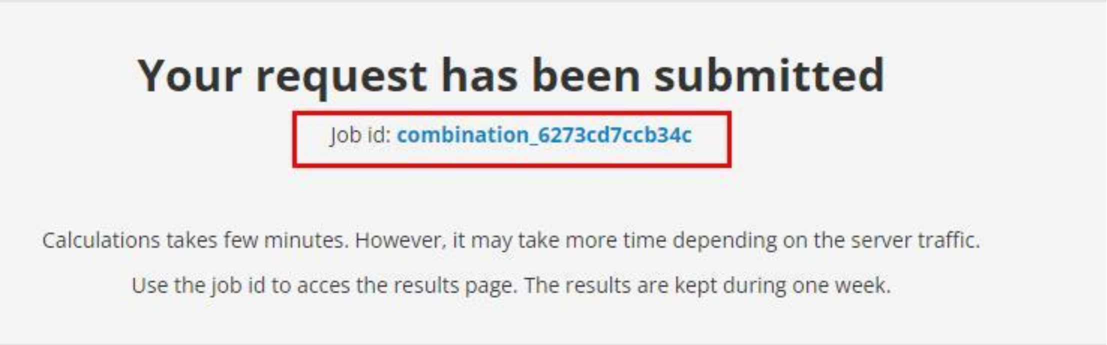

Fig. S3 Website prediction interaction protein successfully submitted

Prediction summary

Protein	Protein	Prediction	Score	Inferred precision	Interaction details
5276	WP_006200394.1	YES	0.503	0.58 ± 0.008	inspect
5276	WP_006199474.1	YES	0.601	0.67 ± 0.009	inspect
3347	WP_006200394.1	YES	0.725	0.73 ± 0.008	inspect
3347	WP_006199474.1	YES	0.889	0.85 ± 0.004	inspect
5902	WP_006199474.1	YES	0.809	0.80 ± 0.009	inspect
5689	WP_006199474.1	YES	0.930	0.85 ± 0.010	inspect
5902	WP_006200394.1	YES	0.702	0.73 ± 0.008	inspect
5689	WP_006200394.1	YES	0.709	0.73 ± 0.008	inspect
3347	WP_006200774.1	N/A	N/A	N/A	No protein features
5276	WP_006200774.1	N/A	N/A	N/A	No protein features
5689	WP_006200774.1	N/A	N/A	N/A	No protein features
5902	WP_006200774.1	N/A	N/A	N/A	No protein features
3347	WP_006200926.1	N/A	N/A	N/A	No interaction signatures
5276	WP_006200926.1	N/A	N/A	N/A	No interaction signatures
5689	WP_006200926.1	N/A	N/A	N/A	No interaction signatures
5902	WP_006200926.1	N/A	N/A	N/A	No interaction signatures
3347	WP_006201247.1	N/A	N/A	N/A	No protein features
5276	WP_006201247.1	N/A	N/A	N/A	No protein features

Fig. S 4 Results of Web site prediction interaction protein

Fig. S5

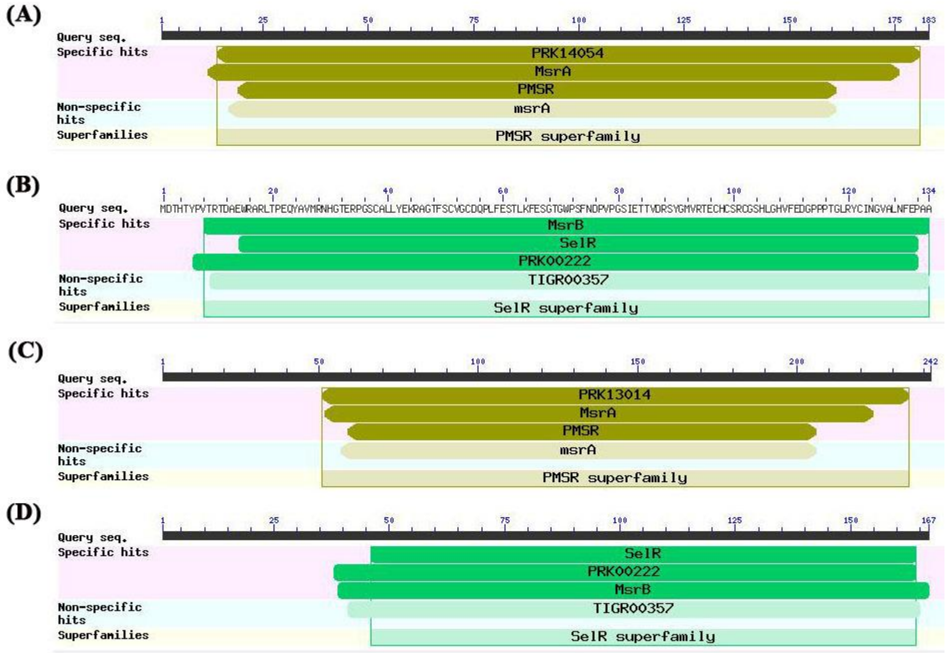

Fig. S5 Conserved domains of *MsrA/B* in *M. huakuii* 7653R. A, conserved domain of MsrA1. B, conserved domain of MsrB1. C, conserved domain of MsrA2. D, conserved domain of MsrB2.

Fig. S6

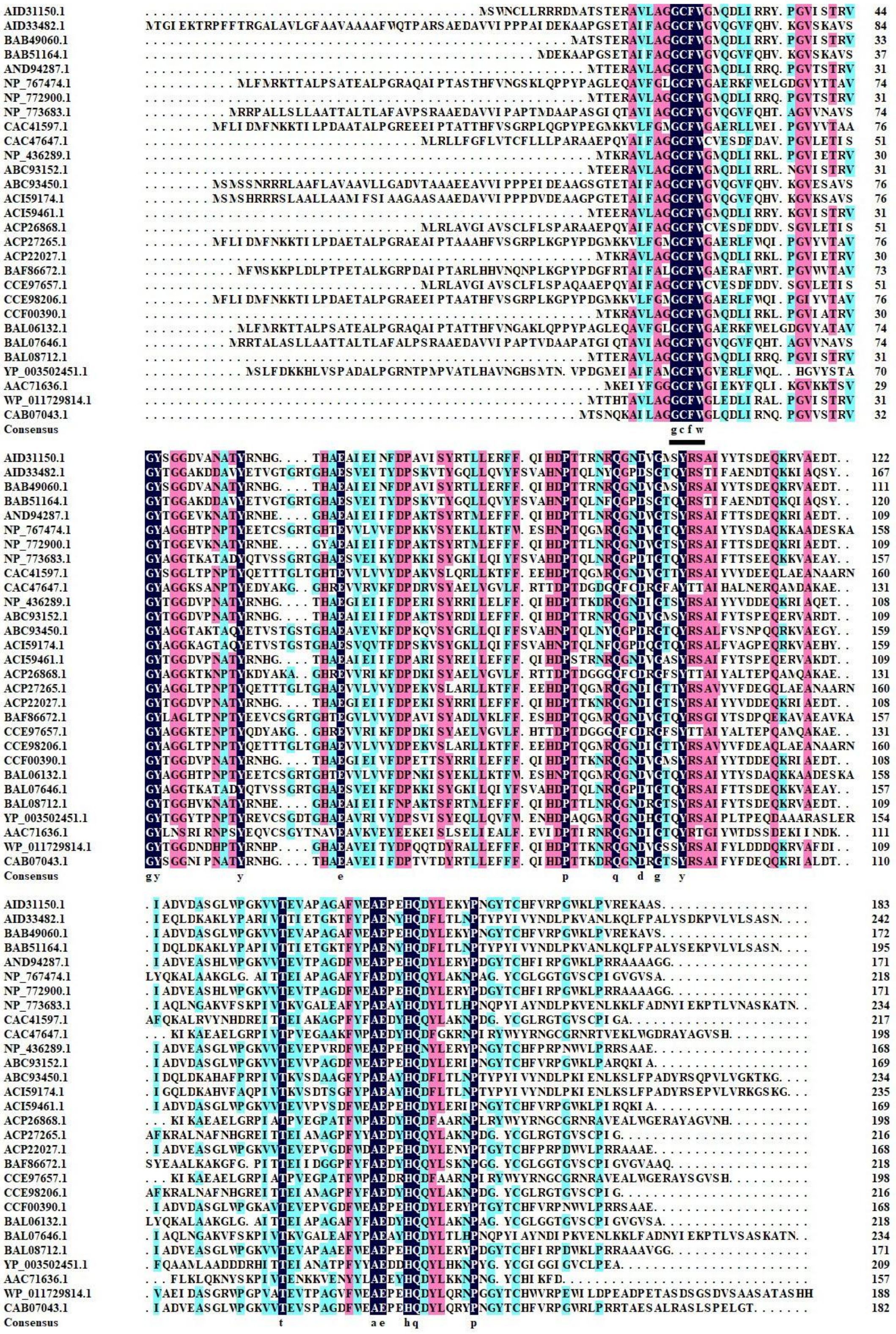
AID31150.1
AID33482.1
BAB49060.1
BAB51164.1
AND94287.1
NP_767474.1
NP_772900.1
NP_773683.1
CAC41597.1
CAC47647.1
NP_436289.1
ABC93152.1
ABC93450.1
ACI59174.1
ACI59461.1
ACP26868.1
ACP27265.1
ACP22027.1
BAF86672.1
CCE97657.1
CCE98206.1
CCF00390.1
BAL06132.1
BAL07646.1
BAL08712.1
YP_003502451.1
AAC71636.1
WP_011729814.1
CAB07043.1
Consensus

Fig. S6 Sequence alignment of MsrAs from different species. The software "DNAMAN" was used for MsrA sequences alignment. MsrA accession numbers were obtained from the NCBI database. AID31150.1 and AID33482.1 from *Mesorhizobium huakuii* 7653R; BAB51164.1 and AND94287.1 from *M. japonicum* MAFF 303099; AND94287.1, NP_772900.1, NP_767474.1 and NP_773683.1 from *Bradyrhizobium diazoefficiens* USDA 110; CAC41597.1, CAC47647.1 and NP_436289.1 from *Sinorhizobium meliloti* 1021; ABC93152.1 and ABC93450.1 from *Rhizobium etli* CFN 42; ACI59174.1 and ACI59461.1 from *R. leguminosarum* bv. trifolii WSM2304; ACP26868.1, ACP27265.1 and ACP22027.1 from *S. fredii* NGR234; BAF86672.1 from *Azorhizobium caulinodans* ORS 571; CCE97657.1, CCE98206.1 and CCF00390.1 from *S. fredii* HH103; BAL06132.1, BAL07646.1 and BAL08712.1 from *B. japonicum* USDA 6; YP_003502451.1 from *Escherichia coli* O55:H7; AAC71636.1 from *Mycoplasma genitalium* G37; WP_011729814 from *Mycolicibacterium smegmatis*; CAB07043.1 from *M. tuberculosis* H37Rv. Conserved motif GCFW in the full-length sequence is underlined.

Fig. S7

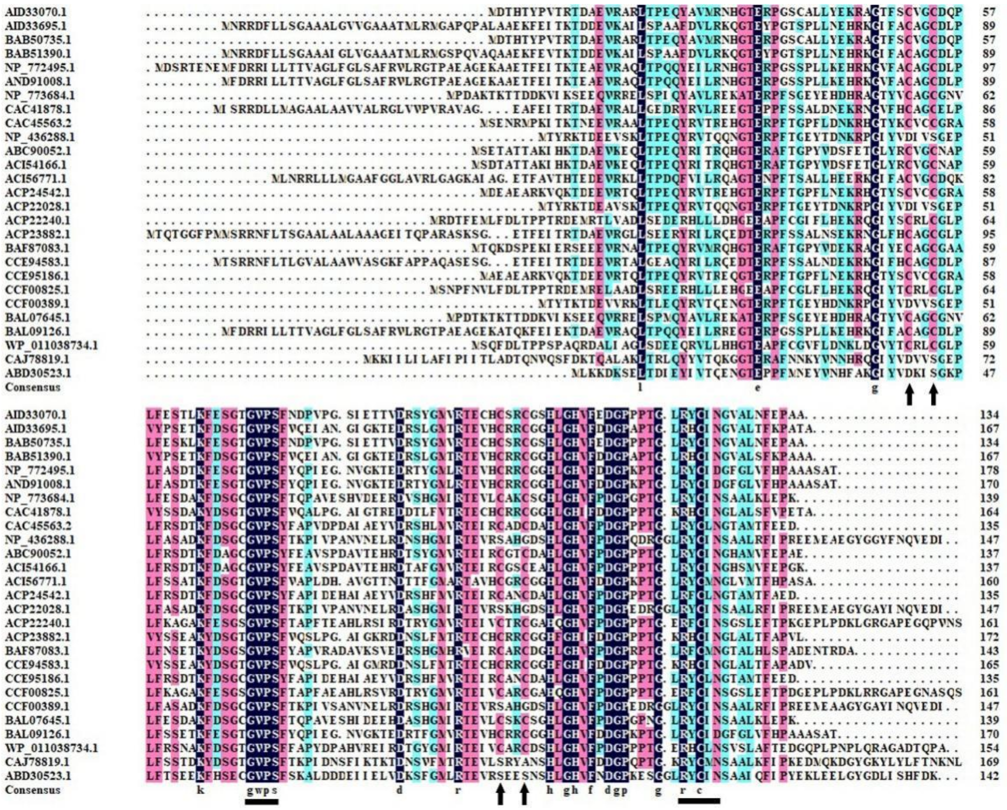

Fig. S7 Sequence alignment of MsrBs from different species. The software “DNAMAN” was used for MsrB sequences alignment. MsrB accession numbers were obtained from NCBI database. AID33070.1 and AID33695.1 from *M. huakuii* 7653R; BAB50735.1 and BAB51390.1 from *M. japonicum* MAFF 303099; NP_772495.1, AND91008.1 and NP_773684.1 from *B. diazoefficiens* USDA 110; CAC41878.1, CAC45563.2 and NP_436288.1 from *S. meliloti* 1021; ABC90052.1 from *R. etli* CFN 42; ACI54166.1 and ACI56771.1 from *R. leguminosarum* bv. Trifolii WSM2304; ACP24542.1, ACP22028.1 ACP23882.1 and ACP22240.1 from *S. fredii* NGR234; BAF87083.1 from *Azorhizobium caulinodans* ORS 571; CCE94583.1, CCE95186.1, CCF00825.1 and CCF00389.1 from *S. fredii* HH103; BAL07645.1 and BAL09126.1 from *B. japonicum* USDA 6; NP_416292.1 from *Escherichia coli* MG1655; WP_011038734.1 from *Xanthomonas campestris*; CAJ78819.1 from *Francisella tularensis* LVS; ABD30523.1 from *Staphylococcus aureus* 8325. Conserved motif GWPS and RXCX in the full-length sequence is underlined. The two arrows indicate the two pairs of Zn^{2+}-conjugating Cys, respectively.

Fig. S8

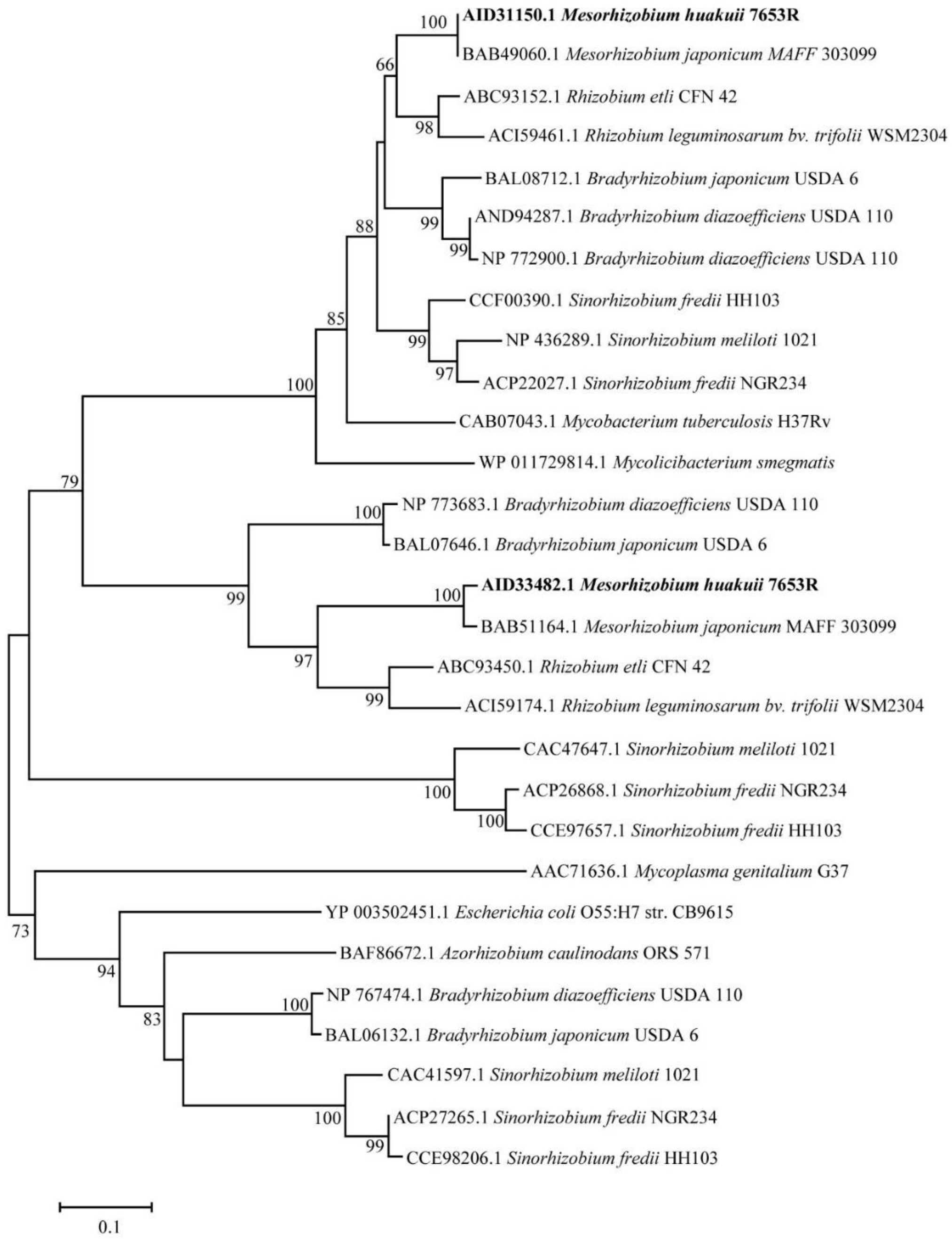

Fig. S8 Phylogenetic trees of MsrAs in different species. Phylogenetic tree of MsrAs in different species. The protein ID of MsrA1 and MsrA2 in *Mesorhizobium huakuii* 7653R are in black. The evolutionary history was inferred using the Neighbor-Joining method. The bootstrap consensus tree inferred from 1000 replicates is taken to represent the evolutionary history of the taxa analyzed. Branches corresponding to partitions reproduced in less than 60% bootstrap replicates are collapsed. Evolutionary analyses were conducted in MEGA6.

Fig. S9

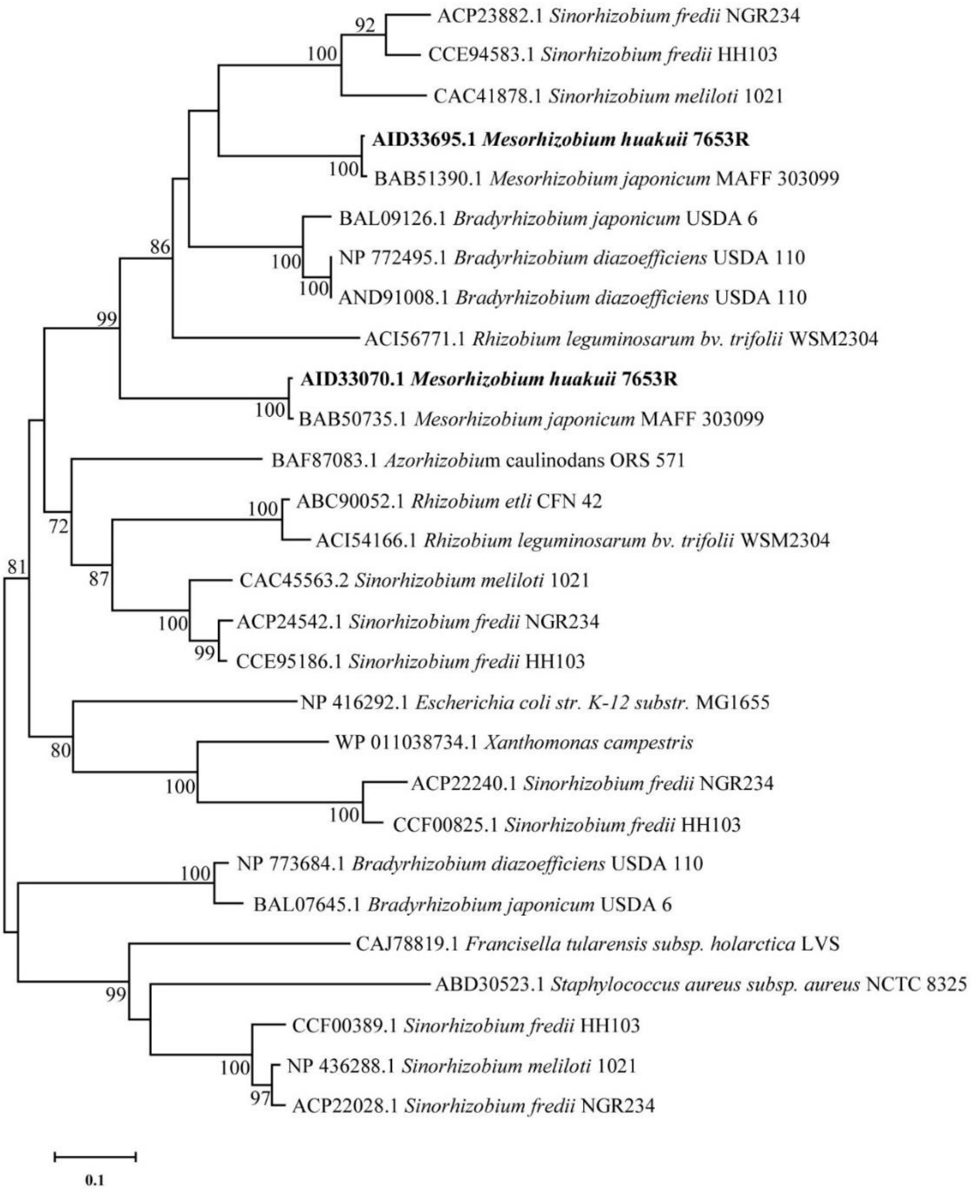

Fig. S9 Phylogenetic trees of MsrBs in different species. Phylogenetic tree of MsrBs in different species. The protein ID of MsrB1 and MsrB2 in *Mesorhizobium huakuii* 7653R are in black. The evolutionary history was inferred using the Neighbor-Joining method. The bootstrap consensus tree inferred from 1000 replicates is taken to represent the evolutionary history of the taxa analyzed. Branches corresponding to partitions reproduced in less than 60% bootstrap replicates are collapsed. Evolutionary analyses were conducted in MEGA6.

Fig. S10

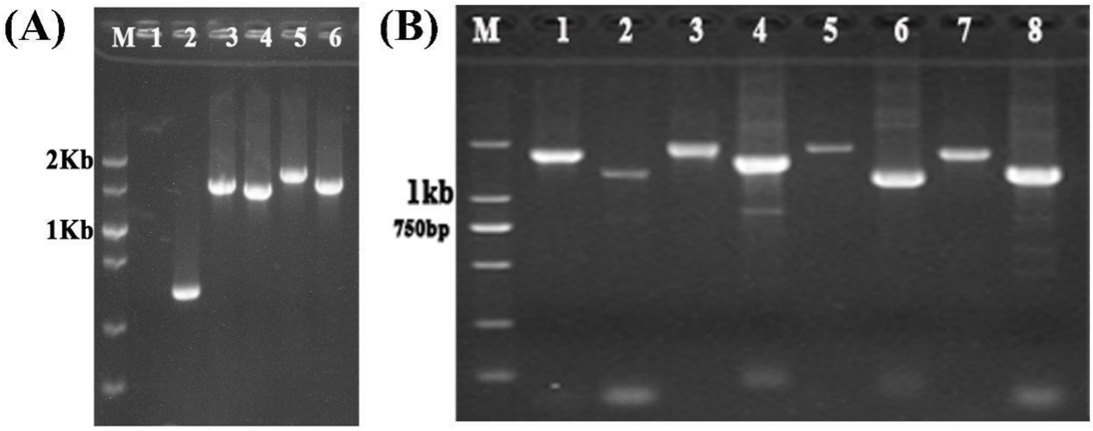

Fig. S10 Verification of *msrA/Bs* overexpression and deletion mutant strains. A, *msrA/Bs* overexpression strains were confirmed by the universal primer PBBR-F/R. M, marker 12, Lane 1-6, indicate strains of WT, WT (empty), *msrA1*OE, *msrB1*OE, *msrA2*OE and *msrB2*OE, respectively. B, *MsrA/Bs* deletion mutant strains were confirmed by the corresponding primers. M, DL2000 ladder. Lane 1, 3, 5, 7, indicate the length of *msrA1*, *msrB1*, *msrA2* and *msrB2* in WT, respectively. Lane 2, 4, 6, 8, indicate the length of deletion mutants of *msrA1Δ*, *msrB1Δ*, *msrA2Δ and msrB2Δ*, respectively. WT indicated *M. huakuii* 7653R, WT (empty) indicated *M. huakuii* 7653R containing the empty overexpression vector. All the validation primers were list in the Table S2.

Fig. S11

(A) The plasmid map of pBBR1MCS-5.

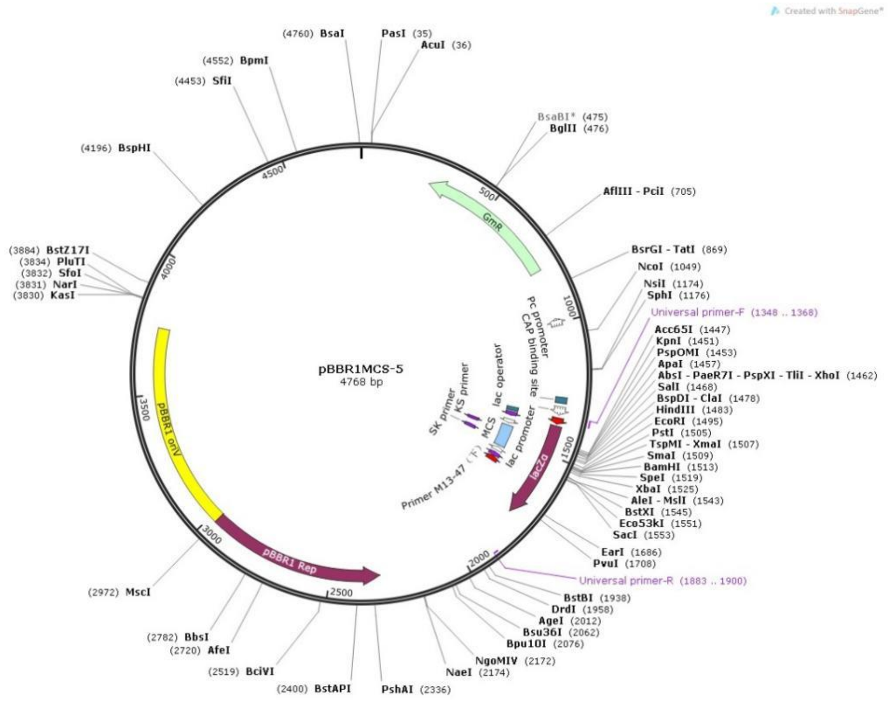

(B)

>MCHK_3347OE

TGTGGAATTGTGAGCGGATAACAATTTCACACAGGAAACAGCTATGACCATGATTACGCCAAGCGCGCAATTAACCCTCA
CTAAAGGGAACAAAAGCTGGGTACCGGGCCCCCCCTCGAGGTCGACGGTATCGATAAGCTTGATATCGAATTCCTAACTC
AATGCCTATGACAAGCCGTCGATCTTCACCGATCTCGATACGCGGTTCGCGCCCTGGATCACCAGGCAGCGCTTGCACGAC
CACGGCATGCTGATGGTTTGGCCAGGGCATGACGTTCCGCCGCAGTTGCAGACATGGCTGGGCAACCTCCCGGTCAAGAC
GGTTCTTTTCAATTGGTCTCGCACGGCGCCGCCGGTGGCAATCAGCTTTGCCGCCATTCCGCCCGGCACGCTGTATCCGCCG
CTTGCCGTCGACAGCCGGACCCTGCATCCGGGCAACTGAAACGGTTCGATCCCCGACCGCGTCGTGATATCGTTCGCTCAT
GGCCGCGTTGTTTCCACGCGACTGGACGAAGCGGACGACAATGCCCATCTATTGGGAAATCCAATTTTGTCCGCGCAATTC
CGGGCGGGAAACCGCTACATGAGCTGGAATTGCCTTTTGAGGAGACGTGACATGGCGACTTCGACCGAACGCGCAGTGCT
TGCCGGTGGCTGTTTCTGGGGCATGCAGGATCTGATACGGCGCTATCCCGGCGTCATCTCCACGCGTGTCGGCTATAGCGG
CGGGGATGTAGCCAACGCCACCTATCGCAACCACGGCACCCATGCCGAAGCCATCGAGATCAATTTCGATCCGGCCGTGA
TCAGCTATCGCACGCTGCTGGAGCGCTTTTTCCAGATCCACGATCCGACGACGCGCAACCGCCAGGGCAACGATGTCGGG
ATGAGCTACCGTTCGGCGATCTACTATACGAGCGACGAGCAGAAACGCGTCGCCGAAGACACCATCGCCGATGTCGATGC
TTCCGGCCTGTGGCCTGGCAAGGTCGTCACGGAAGTCGCGCCGGCCGGCGCCTTCTGGGAAGCCGAGCCCGAGCACCAGG
ATTATCTGGAGAAATATCCAAACGGCTACACCTGCCACTTCGTCAGGCCGGGCTGGAAACTGCCGGTTCGCGAAAAGGCC
GCATCCTAAGGATCCACTAGTTCTAGAGCGGCCGCCACCGCGGTGGAGCTCCAATTCGCCCTATAGTGAGTCGTATTACGC
GCGCTCACTGGCCGTCGTTTTACAACGTCGTGACTGGGAAAACCCTGGCGTTACCCAACTTAATCGCCTTGCAGCACATCC
CCCTTTCGCCAGCTGGCGTAATAGCGAAGAGGCCCGCACCGATCGCCCTTCCCAACAGTTGCGCAGCCTGAATGGCGAAT
GGAAATTGTAAGCGTTAATATTTTGTTAAAATTCGCGTTAAATTTTTGTTAAATCAGCTCATTTTTTAACCAATAGGCCGAC
TGCGATGAGTGGCAGGGCGGGGCGTAATTTTTTTAAGGCAGTTATTGGTGCCCTTAAACGCCTGGTGCTACGC

>MCHK_5276OE

TGTGGAATTGTGAGCGGATAACAATTTCACACAGGAAACAGCTATGACCATGATTACGCCAAGCGCGCAATTAACCCTCA
CTAAAGGGAACAAAAGCTGGGTACCGGGCCCCCCCTCGAGGTCGACGGTATCGATAAGCTTGATATCGAATTCCTAACTA
TCTCCACGCCGACGACCGCCAATGGATCATGCGGCCGCATCCCTCGATCGTCAGTTGGCAAGGCGAGACGCTCGTCCTCAA
TCCGTCGCTCACGCTCGTCCGCTGCGGCGGCCATTTTGCCGGCAGCCAGGTGCTGCACTGGCAGCGCGAGGGCGGCAACG
CCCTACTGACCGGCGACACGGTGCAGGTGACGCCAACGCGCCGCCATGTCAGCTTCATGTACAGCTATCCCAACCAGATA
CCGCTCAACGCGGCGGCCGTGCGCCGCATAGCGGCGGTATTGGAACCATTCGCCTTCGACGATATCCGCGGGGCATGGTG
GGACCTGAACATCATCGGCGGTGGCAAGACGGCCCTGGCCACCTCTGTCGCGCGCTATCTGGCGGCGATAGGATGATGAT
CGCGAGCGTAAATGCGGGCAAACCTTGTAACAAATGCCTATTTGTTGCGAATATGAGGTTGCAACAAAGAACGAGCCGCC
AAGGCTGAGGAGATCAAATGGACACCCACACTTACCCCGTCACCCGCACCGATGCCGAATGGCGCGCCCCGGCTGACGCCG
GAGCAATATGCGGTCATGCGCAATCACGGCACCGAGCGGCCGGGCAGCTGCGCTTTGCTCTACGAGAAGCGCGCCGGCAC
ATTTTCCTGCGTCGGCTGCGACCAGCCGCTGTTCGAATCCACGCTGAAGTTCGAGAGCGGCACCGGCTGGCCGAGCTTCAA
CGACCCGGTGCCCGGCTCGATCGAGACCACCGTCGACCGCAGCTACGGCATGGTCCGCACCGAGTGCCATTGCTCGCGCT
GCGGCAGCCATCTCGGCCACGTCTTCGAGGACGGCCCGCCGCCGACAGGTCTGCGCTATTGCATCAACGGCGTGGCGCTTA
ATTTCGAGCCGGCGGCCTGAGGATCCACTAGTTCTAGAGCGGCCGCCACCGCGGTGGAGCTCCAATTCGCCCTATAGTGAG
TCGTATTACGCGCGCTCACTGGCCGTCGTTTTACAACGTCGTGACTGGGAAAACCCTGGCGTTACCCAACTTAATCGCCTT
GCAGCACATCCCCCTTTCGCCAGCTGGCGTAATAGCGAAGAGGCCCGCACCGATCGCCCTTCCCAACAGTTGCGCAGCCTG
AATGGCGAATGGAAATTGTAAGCGTTAATATTTTGTTAAAATTCGCGTTAAATTTTTGTTAAATCAGCTCATTTTTTAACCA
ATAGGCCGACTGCGATGAGTGGCAGGGCGGGGCGTAATTTTTTTAAGGCAGTTATTGGTGCCCTTAAACGCCTGGTGCTAC
GC

***>MCHK_5689*OE**

TGTGGAATTGTGAGCGGATAACAATTTCACACAGGAAACAGCTATGACCATGATTACGCCAAGCGCGCAATTAACCCTCA
CTAAAGGGAACAAAAGCTGGGTACCGGGCCCCCCCTCGAGGTCGACGGTATCGATAAGCTTGATATCGAATTCCTAAACC
AAGTACCTGACCCTGCCGGCGCTCGCCTTCGCGCTCTCGGCCGCCGTTTTCGTCGCCGGCGCCCGTGCCGACGACGCGACC
AACGCCATGAAGCCCGCCAATGCGATGGCCACCGACGCCATGAAGCCGGCGACGGATGCGATGAAGCCGGCGGATGCCA
TGAAGCCCGCAACCGACGCGATGAAGCCGGCCGACCCGATGGCCACCAATGCGATGAAGCCAGCCACCGACGCGATGAA
GCCAGCGCAGTAAGCAGGTCGGCTGCCCCTCACCCAGCCTCCGCTTCGCTCGCCTGACCGTTCCGGGGCGAACGGCGCCA
ACTTTCTTCTCCCCCTCGGGGAGAAGGTGGCCCGAAGGGCCGGATGAGGGGGAGTGCCGGCGACAGGAGCCGCCCGCAC
GGCAAATTGAGACCTCTTGGCGGCTTGCGTCGTATATATTGGAACAACAACAGGAGATCAGAACATGACCGGTATCGAAA
AGACACGGCCGTTCTTTACCCGCGGCGCGCTGGCCGTGCTCGGTTTTGCCGCGGTGGCGGCGGCCGCCTTCTGGCAGACCC
CGGCGCGCTCCGCCGAGGACGCGGTGGTGATCCCGCCGCCGGCAATTGACGAAAAGGCCGCCCCGGGCAGCGAGACGGC
GATCTTCGCCGGCGGCTGCTTCTGGGGCGTGCAGGGCGTGTTCCAGCACGTCAAGGGCGTCAGCAAGGCCGTCTCCGGCTA
TACCGGCGGCGCCAAGGATGACGCCGTCTACGAGACCGTCGGCACCGGCCGCACCGGCCATGCCGAATCCGTCGAGATCA
CCTACGACCCGTCGAAGGTGACCTACGGCCAGCTGCTGCAGGTCTATTTCTCGGTCGCCCACAATCCGACGCAGCTGAACT
ACCAGGGACCGGATTCCGGCACCCAGTACCGCTCGACGATCTTCGCCGAGAACGACACGCAAAAGAAGATCGCGCAGAG
CTATATCGAGCAGCTCGACAAGGCCAAGCTCTACCCGGCACGAATCGTCACCACGATCGAGACCGGCAAGACCTTCTATC
CGGCCGAGAACTACCACCAGGATTTCCTGACGCTGAACCCGACCTATCCCTACATCGTCTACAACGACCTGCCCAAGGTGG
CGAACCTGAAGCAGCTGTTCCCGGCGCTGTACAGCGACAAGCCGGTGCTGGTTCTGTCGGCCAGCAATTGAGGATCCACT
AGTTCTAGAGCGGCCGCCACCGCGGTGGAGCTCCAATTCGCCCTATAGTGAGTCGTATTACGCGCGCTCACTGGCCGTCGT
TTTACAACGTCGTGACTGGGAAAACCCTGGCGTTACCCAACTTAATCGCCTTGCAGCACATCCCCCTTTCGCCAGCTGGCG
TAATAGCGAAGAGGCCCGCACCGATCGCCCTTCCCAACAGTTGCGCAGCCTGAATGGCGAATGGAAATTGTAAGCGTTAA
TATTTTGTTAAAATTCGCGTTAAATTTTTGTTAAATCAGCTCATTTTTTAACCAATAGGCCGACTGCGATGAGTGGCAGGGC
GGGGCGTAATTTTTTTAAGGCAGTTATTGGTGCCCTTAAACGCCTGGTGCTACGC

>*MCHK_5902*OE

TGTGGAATTGTGAGCGGATAACAATTTCACACAGGAAACAGCTATGACCATGATTACGCCAAGCGCGCAATTAACCCTCA
CTAAAGGGAACAAAAGCTGGGTACCGGGCCCCCCCTCGAGGTCGACGGTATCGATAAGCTTGATATCGAATTCCTAAGCT
GCTCAAGCCCGAGAACAAGGACAAGCTGACGAAGGTCCTGACCTGCCATGTCATCGGCGCCAAGGCGATGGCGGCGGAT
GTGGCCTCGATGGCCAAGGCCGACGGCGGCACGCACAAGGTCAAGACCGTCGGCGGCTGCGAGCTTTCCCTGAAGGCCGA
GGGCGGCAAGGTCACCGTTACCGACGAGAATGGCAATGTCGCCAATGTGACGATCGCCGATGTCGAGCAGTCCAACGGTG
TCATCCACGTCATCGACAAGGTTCTTTTGCCGAAGATGTAACAAGGGGCCAGCCGCGGACGAACCTGCGGCAAGAGCTCT
CCGCCGATTGCGTCGTCCCGCTCCCCTGATCCGCAATCGACACGCACGCTCCGCGCCGCCCACAGCCAACTGTGGAACGGC
GCGGAGTGTCGTTTGGGGGCAACCGTCACCGGCTTTTGATTTTGGCGTGGCGGTATAGTTTGGCAAAAGGAACAACCGGA
GGAATGGTCATGAACCGTCGCGACTTTCTTTTGAGCGGTGCCGCCGCCCTTGGCGTCGTCGGGGCCGCCGCAACCATGCTG
CGCATGGGCGCTCCGCAGCCCGCGCTGGCGGCCGAAAAATTCGAGATCACCAAGACGGATGACGAGTGGAAAGCCATCCT
GTCGCCTGCCGCCTTCGACGTGCTGCGCAAGCAAGGCACGGAATATCCGGGCACCAGCCCGCTGCTCAACGAGCACCGCA
AGGGTATCTTCGCCTGTGCCGGCTGCGACCTGCCGGTCTACCCCTCTGAGACGAAATTCGATTCCGGCACCGGCTGGCCGA
GCTTCTGGCAGGAGATCGCCAACGGCATCGGCAAGACCGAGGACAGGTCGCTCGGCATGACCCGCACCGAGGTGCATTGC
CGCCGCTGCGGCGGCCATCTCGGCCACGTCTTCGACGACGGTCCGGCGCCGACCGGCCTGCGCCACTGCATCAACGGCGT
GGCGCTGACCTTCAAGCCGGCCACGGCCTGAGGATCCACTAGTTCTAGAGCGGCCGCCACCGCGGTGGAGCTCCAATTCG
CCCTATAGTGAGTCGTATTACGCGCGCTCACTGGCCGTCGTTTTACAACGTCGTGACTGGGAAAACCCTGGCGTTACCCAA
CTTAATCGCCTTGCAGCACATCCCCCTTTCGCCAGCTGGCGTAATAGCGAAGAGGCCCGCACCGATCGCCCTTCCCAACAG
TTGCGCAGCCTGAATGGCGAATGGAAATTGTAAGCGTTAATATTTTGTTAAAATTCGCGTTAAATTTTTGTTAAATCAGCTC
ATTTTTTAACCAATAGGCCGACTGCGATGAGTGGCAGGGCGGGGCGTAATTTTTTTAAGGCAGTTATTGGTGCCCTTAAAC
GCCTGGTGCTACGC

Fig. S11 The overexpression plasmid map and the sequence results of PCR product. A, the plasmid map of pBBR1MCS-5, the position of universal primer F/R were marked purple. B, the sequence information of PCR product with the universal primer F/R for the *msrA1*OE, *msrB1*OE, *msrA2*OE and *msrB2*OE, respectively. The background of their promoters and open reading frames (ORFs) were marked yellow. The universal primer F/R sequence were marked red. All the amplified product of real-time PCR primers were marked by underline.

Fig. S12

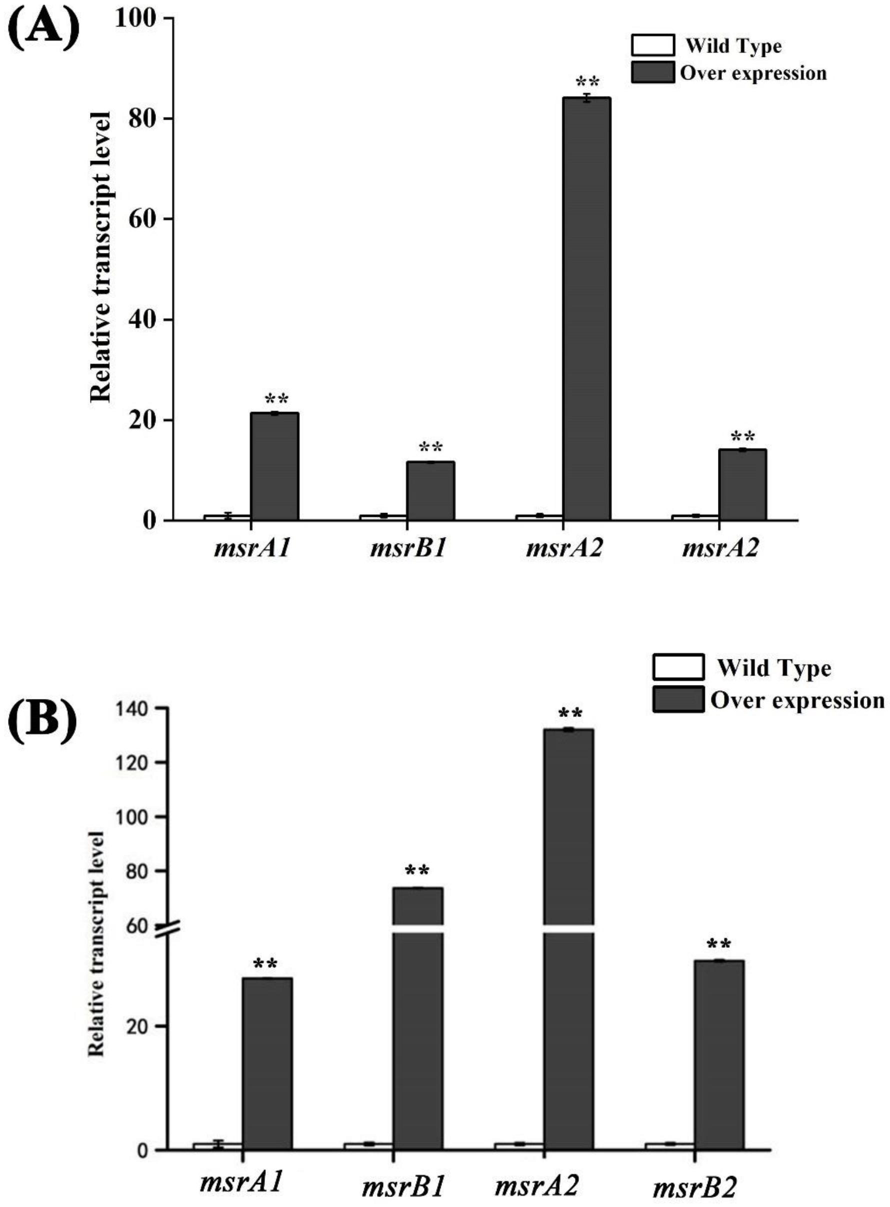

Fig. S12 Relative transcript level of *msrs* in overexpressing strains and nodules. (A), Relative transcript level of *msrs* in overexpressing strains. The total RNA was extracted from *M. huakuii* 7653R (Wild type), *msrA1*OE, *msrB1*OE, *msrA2*OE and *msrB2*OE, respectively. (B), Relative transcript levels of *msrs* in overexpression nodules. The nodules were collected from plants inoculated with *M. huakuii* 7653R (Wild type), *msrA1*OE, *msrB1*OE, *msrA2*OE and *msrB2*OE at 30 dpi, respectively. The relative transcript level was determined by qRT-PCR and normalized to that of *rnpB*. ** ($p < 0.01$) indicate significant differences and extremely significant differences between the two groups as determined by students *t* test.

Fig. S13

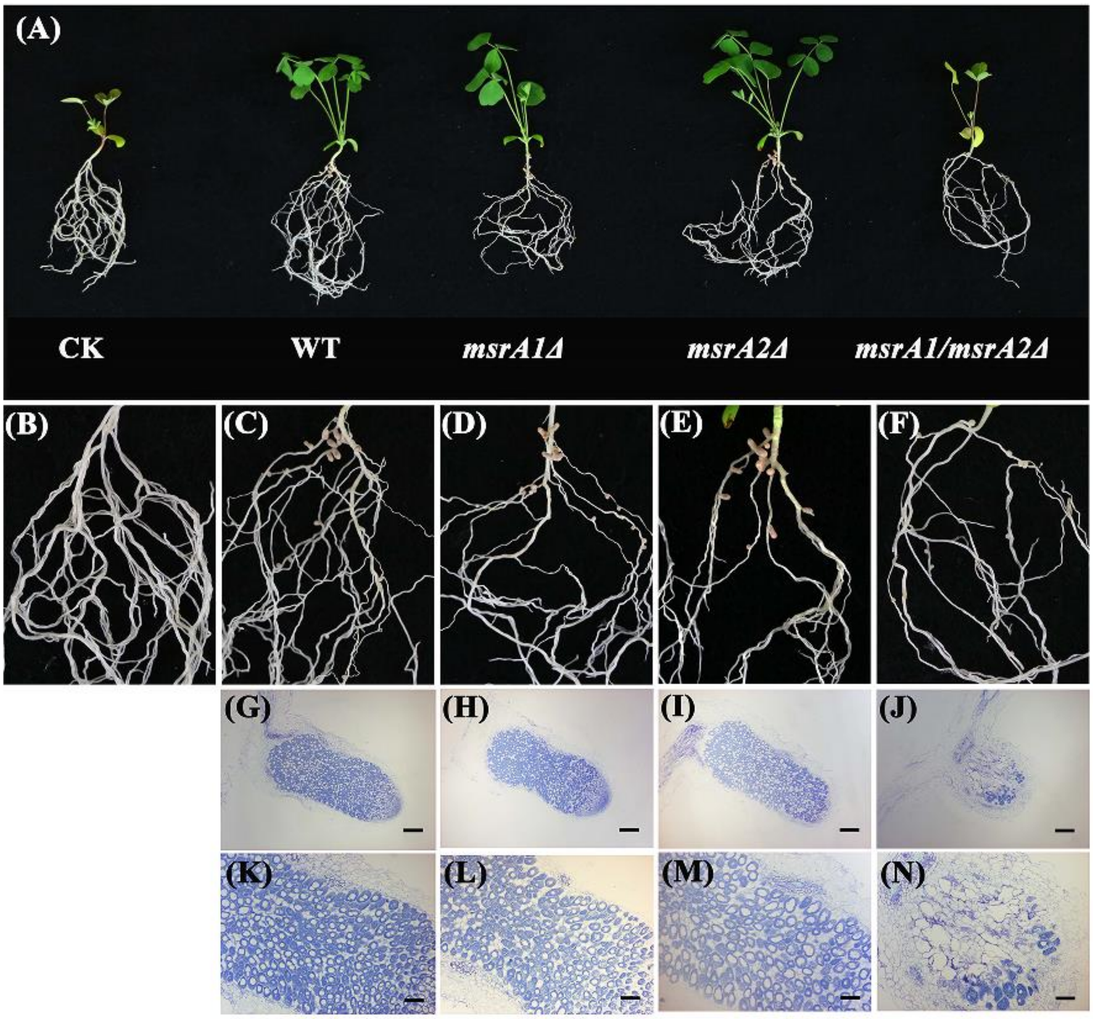

Fig. S13 Symbiotic Phenotypes and Longitudinal Paraffin Sections Induced by *msrA1/A2* double mutants. (A), Whole-plant phenotypes at 27 days post-inoculation (dpi): from left to right, non-inoculated control, wild-type (WT), *msrA1Δ*, *msrA2Δ*, and *msrA1/B2Δ*. (B), Representative root system of the non-inoculated control. (C–F), Representative images of roots and nodules from plants inoculated with WT, *msrA1Δ*, *msrA2Δ*, and *msrA1/A2Δ*, respectively. (G, H, I, J), Nodules induced by wild-type (WT), *msrA1Δ*, *msrA2Δ* and *A1/A2Δ*, respectively. (K, L, M, N), Magnified views of the nitrogen-fixing zones corresponding to panels, G, H, I and J, respectively. All images were taken and nodules were collected at 27 dpi. Scale bars: 500 μm (G–J); 200 μm (K–N).

Acid Minimal Salts (AMS) Medium

Ingredients

0.5 ml	1M K_2HPO_4 (c/r)	Di-potassium hydrogen orthophosphate anhydrous (0.5 mM)
0.5 g	$MgSO_4.7H_2O$	Magnesium sulphate (2 mM)
0.2 g	NaCl	Sodium Chloride
4.19 g	MWT 209.3	MOPS (20 mM)
1000 mlGDW		
1 ml	Solution A	
2 ml	Solution B	Do not store for more than 1 week
1 ml	Solution C	Added aseptically after sterilising

UNLESS dispensing small volumes in which case add solution C before sterilising.

Method

Dissolve all chemical ingredients in GDW then adjust pH to 7. Add solutions A and B. Check pH.

UNLESS STATED THERE IS NO CARBON OR NITROGEN SOURCE.
Autoclave at 15 psi/121°C
Add solution C aseptically after sterilizing.

Rhizobium Solution A

Ingredients:

15 g	EDTA-Na_2
0.16 g	$ZnSO_4.7H_2O$ (Zinc sulphate heptahydrate)
0.2 g	$NaMoO_4$ (Sodium molybdate di-hydrate)
0.25 g	H_3BO_3 (Boric acid)
0.2 g	$MnSO_4.4H_2O$ (Manganese sulphate tetrahydrate)
0.02 g	$CuSO_4.5H_2O$ (Copper sulphate pentahydrate)
1mg	$CoCl_2.6H_2O$ (Cobalt chloride hexahydrate)

(Dissolve 100mg in 100ml GDW and add 1ml)

Make up to 1L in GDW

Method:

Dissolve each ingredient in turn before adding the next.Make up w/v to 1L with GDW.
Store at 4°C.

Rhizobium Solution B

Ingredients:

1.28 g	$CaCl_2.2H_2O$ (87.1 mM) (final after addition to main medium 0.17

mM)0.33 g	$FeSO_4.7H_2O$
100 ml	GDW

Method:

Dissolve each ingredient in 50mls water then combine.Store at 4°C for no more than 1 week.

Rhizobium Solution C

Ingredients:

1 g	Thiamine hydrochloride
2 g	D-Pantothenic acid Ca salt
1 mg	Biotin (Dissolve 100mg in 1000ml GDW and add 10 ml store rest of biotin at -20°C)

Make up to 1L with GDW

Method:

Dissolve each ingredient in turn before adding the next.Make w/v to 1L with GDW

FILTER STERILISE and store at 4°C Add aseptically to media at 1ml per liter.
